# Defining the Surface Oxygen Threshold That Switches the Interaction Mode of Graphene Oxide with Bacteria

**DOI:** 10.1021/acsnano.2c10961

**Published:** 2023-02-26

**Authors:** Zhiling Guo, Peng Zhang, Changjian Xie, Evangelos Voyiatzis, Klaus Faserl, Andrew J. Chetwynd, Fazel Abdolahpur Monikh, Georgia Melagraki, Zhiyong Zhang, Willie J. G. M. Peijnenburg, Antreas Afantitis, Chunying Chen, Iseult Lynch

**Affiliations:** † School of Geography, Earth and Environmental Sciences, University of Birmingham, Edgbaston, Birmingham B15 2TT, United Kingdom; ‡ Department of Environmental Science and Engineering, University of Science and Technology of China, Hefei 230026, China; § School of Life Sciences and Medicine, Shandong University of Technology, Zibo 255000, Shandong, China; ∥ Nanoinformatics Department, NovaMechanics Ltd., Nicosia, 1065, Cyprus; ⊥ Institute of Medical Biochemistry, Medical University of Innsbruck, 6020 Innsbruck, Austria; # Department of Environmental & Biological Sciences, University of Eastern Finland, P.O. Box 111, Joensuu, FI-80101, Finland; ∇ Key Laboratory for Biological Effects of Nanomaterials and Nanosafety, Institute of High Energy Physics, Chinese Academy of Sciences, Beijing 100049, China; ◆ School of Nuclear Science and Technology, University of Chinese Academy of Sciences, Beijing 100049, China; ¶ Institute of Environmental Sciences (CML), Leiden University, Einsteinweg 2, 2333 CC Leiden, The Netherlands; ◇ National Institute of Public Health and the Environment (RIVM), Center for Safety of Substances and Products, 3720 BA Bilthoven, The Netherlands; ■ CAS Center for Excellence in Nanoscience and CAS Key Laboratory for Biomedical Effects of Nanomaterials and Nanosafety, National Center for Nanoscience and Technology of China, Beijing 100190, China; ○ Research Unit of Nanoscience and Technology, Chinese Academy of Medical Sciences, Beijing 100039, China; ● GBA National Institute for Nanotechnology Innovation, Guangdong 510700, China; △ Research Unit of Nanoscience and Technology, Chinese Academy of Medical Sciences, Beijing 100021, China

**Keywords:** graphene oxide, surface oxygen content, oxidative potential, membrane damage, antibacterial activity, interaction mode

## Abstract

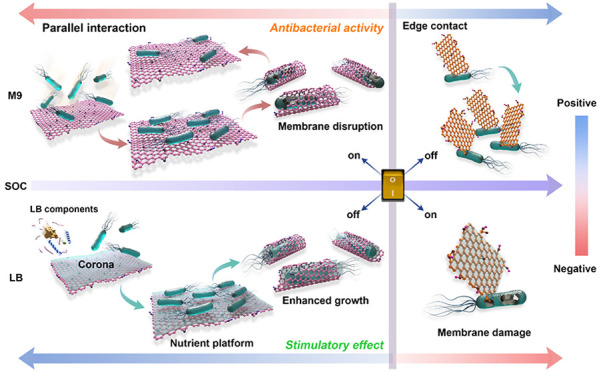

As antimicrobials, graphene materials (GMs) may have advantages over traditional antibiotics due to their physical mechanisms of action which ensure less chance of development of microbial resistance. However, the fundamental question as to whether the antibacterial mechanism of GMs originates from parallel interaction or perpendicular interaction, or from a combination of these, remains poorly understood. Here, we show both experimentally and theoretically that GMs with high surface oxygen content (SOC) predominantly attach in parallel to the bacterial cell surface when in the suspension phase. The interaction mode shifts to perpendicular interaction when the SOC reaches a threshold of ∼0.3 (the atomic percent of O in the total atoms). Such distinct interaction modes are highly related to the rigidity of GMs. Graphene oxide (GO) with high SOC is very flexible and thus can wrap bacteria while reduced GO (rGO) with lower SOC has higher rigidity and tends to contact bacteria with their edges. Neither mode necessarily kills bacteria. Rather, bactericidal activity depends on the interaction of GMs with surrounding biomolecules. These findings suggest that variation of SOC of GMs is a key factor driving the interaction mode with bacteria, thus helping to understand the different possible physical mechanisms leading to their antibacterial activity.

## Introduction

The antibacterial activity of graphene materials (GMs) was reported as early as 2010.^1^ Extensive research has been done to explore the antimicrobial performance of graphene and its derivatives such as graphene oxide (GO) and reduced GO (rGO).^2,3^ The antibacterial properties of GMs can be used for a variety of purposes. For example, graphene modified antibacterial fabrics^4,5^ for maternity garments can prevent microbial growth on the fabric surface. Graphene-coated nonwovens have been used to produce antibacterial masks.^6^ Graphene-based membranes have been extensively studied for water treatment not only because of their ultrafast water transport but also because of their antifouling activity.^7^ In spite of this, translation from laboratory findings to practical application still has a long way to go. In addition to concerns over the potential environmental health and human impacts of graphene,^8^ the mechanisms of the toxic impacts of graphene on microbes remain under debate,^9^ hindering the progress in developing antibacterial GMs as well as in understanding the environmental safety of GMs.

It has been proposed that the antimicrobial activity of GMs involves both chemical and physical interactions.^10^ A major indeterminacy is on the physical interaction mode of graphene with bacteria. A widely accepted viewpoint is that graphene penetrates the cell membranes via its sharp edges.^11,12^ It has been shown by computational simulations that the perpendicular orientation of graphene on the cell membrane can penetrate the cell membrane and extract phospholipids which leads to membrane damage.^8^ Experimental findings also suggest that antibacterial activities can be enhanced through vertical alignment of graphene on a substrate.^13^ A wrapping mechanism is also proposed in which large graphene sheets may entrap the bacteria, preventing the acquirement of nutrients and thus inhibiting bacterial proliferation.^14^ However, other studies found that substrates coated with parallelly arranged graphene (so that no sharp edge contact or wrapping is possible) also kill bacteria,^15^ whereas it was also found that in suspension the largely relies on the area of the exposed GM basal plane.^16^ This finding may suggest that perpendicular orientation of GMs on bacteria is not a necessity for killing bacteria and highlights the critical role of the basal surface of GMs in their antibacterial activity.

The surface oxygen content (SOC) is a key parameter determining the surface properties of GMs (e.g., surface charge and hydrophilicity) and thus their interaction with biological organism. For example, hydrophobic interaction and van der Waals attraction between graphene and lipid membrane has been proposed as the driving force for the early stage of graphene penetration into lipid membrane.^11^ Variation of SOC may thus greatly affect this interaction mode due to the change in hydrophobicity of the GMs surface. However, our investigation of the literature on the antibacterial activity of pristine GMs shows that nearly 70% of papers did not report the SOC (Figure S1). This is also evident in studies comparing the antibacterial activity of GO and rGO or graphene, with ∼60% of these not reporting the SOC (Table S1), rendering these results incomparable. Given the crucial role of SOC in controlling the surface properties, we hypothesize that the inconsistency in SOC might be a key factor leading to contradictory opinions on the physical mechanism of antimicrobial action of GMs. Indeed, a preliminary study has given some hints, showing that GO caused higher loss of membrane integrity than rGO. However, unlike rGO, the membrane damage caused by GO was dependent on surface area rather than the total edge length,^16^ suggesting that piercing of the membrane is not a main mechanism of toxicity for GO.

Here, we fabricated a series of GO and rGO materials with different SOC and compared their antibacterial performance, by evaluating the total cell growth, biofilm formation as well as oxidative stress in two representative media. Then we evaluated their physical interactions with bacteria by SEM and a liposome leakage assay. Molecular dynamic (MD) simulations were exploited to understand the GMs–lipid membrane interaction and explore the potential mechanism of the different interactions. We observed an interesting pattern of interaction modes of GMs with bacterial cells, which is controlled by a SOC switch (a threshold value). A slight change of SOC can lead to the shift of interaction modes between parallel and perpendicular contact. We found that it is the different interaction modes that lead to distinct antibacterial activity. The interaction mode is highly related to the rigidity of GMs. GO with high SOC is very flexible and thus tends to attach and wrap bacteria with the basal plane, while rGO with its low SOC is freestanding due to the high rigidity and thus can contact the bacteria edge-wise. Formation of a protein corona on the GO surface can further affect their bactericidal ability by changing the surface chemistry. Our results not only provide fundamental understanding of the interaction of suspended GMs with bacterial cell membranes and contribute to addressing the recently untouched in GM antibacterial studies but also will help to design safer GMs, through clarifying the role of SOC and the acquired corona.

## Results and Discussion

### Antibacterial Activity of GMs Controlled by an SOC Threshold

A library of GO and rGO (G1, G2, G3, G4, G5, and G6) with different SOCd (0.346, 0.336, 0.313, 0.296, 0.263, and 0.078, respectively) but similar average lateral sizes and thicknesses was prepared (see detailed characterization in Figured S2–S8, Table S2 and S3). All the GMs showed no impurities (Figures S4, S6, and S8), ensuring that SOC was the only parameter varying. The antibacterial activity of the GMs against *Escherichia coli* (*E. coli*, Gram-negative bacteria) and *Staphylococcus aureus* (*S. aureus*, Gram-positive bacteria) was examined.

**Figure 1:**
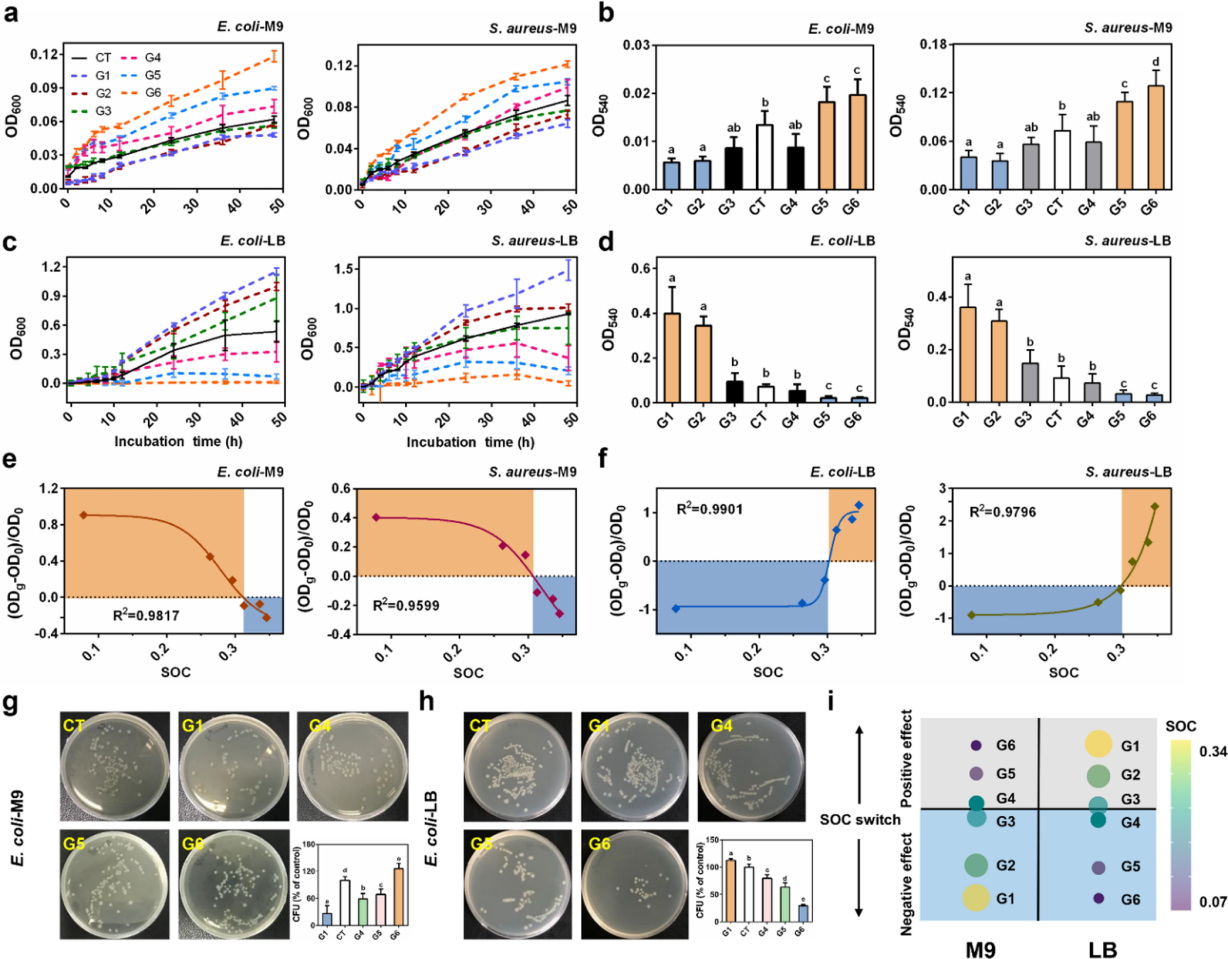
Antibacterial performance of GMs with different surface oxygen content (SOC) against *E. coli* or *S. aureus*in M9 or LB medium. (a, c) Total cell growth (OD_600_) of *E. coli* or *S. aureus* after exposure to 100 mg/L GMs in M9 (a) or LB medium (c) for 48 h (*n* = 6, mean ± s.d.). (b, d) Bacterial biofilm formation (OD_540_) measured by a crystal violet staining method after interaction with GMs in M9 (b) or LB medium (d) for 48 h. Different lowercase letters indicate significant difference at *p* < 0.05 (*n* = 6, mean ± s.d.). (e, f) Logistic regression analysis of the SOC with the percent of loss of total cell viability (OD_600_) in M9 (e) or LB (f) medium. Y axis is the percent of loss of viability of bacterial cells. OD_0_ indicates OD_600_ values in control group. OD_g_ indicates OD_600_ values after exposure to GM suspension for 48 h. The SOC switch was defined as the SOC value where the *Y*-axis value is zero. (g, h) Photographs of colony development on plates and relative numbers of viable *E. coli* colonies formed after 3 h of incubation with GMs in M9 (g) or LB (h) medium (*n* = 6, mean ± s.d.). (i) Bubble plot schematic illustration of the SOC switch controlling the antibacterial activity of GMs. The size of the bubble represents the amount of SOC, with larger bubbles corresponding to higher SOC.

We first performed a static suspension assay, which allowed the formation of a biofilm. Our results showed an SOC-dependent response of both bacteria to GMs in M9 (minimal) medium (Figure 1a,b). G1 and G2, with relatively high SOC, exhibited antibacterial activities. The effects of G3 and G4 were not significant. In contrast, G5 and G6 with lower SOCs showed positive (stimulatory) effects on bacterial growth. We further examined whether a clear difference can also be observed in LB medium, the components of which can potentially adsorb to the surface of GMs and thus can affect their antibacterial activity. We found that the antibacterial performance of the GMs was entirely reversed in LB broth compared with that in M9 medium, with G1 and G2 showing growth enhancing effects while G5 and G6 present antibacterial effects (Figure 1c,d). G3 and G4 showed insignificant effects here also. These results suggest that there is an SOC threshold value which GMs must reach to become active in killing bacteria. By performing logistic regression analysis on the correlation of SOC with the percent of loss of viability (Figures 1e,f and S9), the threshold SOC value was determined to be ∼0.3, corresponding to an atomic percent of O of 30% of the total atoms as measured by X-ray photoelectron spectroscopy (XPS).

Confocal laser scanning microscopy (CLSM) imaging of the bacterial biofilms (Figure S10) and the plate-counting colony formation assay (Figure 1g,h) also showed similar results, with the antibacterial activity gradually decreasing in M9 medium with decreasing SOC, while in LB medium a completely opposite trend was observed. All these data suggest that the antibacterial activity is controlled by an SOC switch, and further affected by the composition of the culture medium. The action of the switch is illustrated schematically in Figure 1i. The GMs showed bactericidal activity in M9 when the SOC > 0.3; when the SOC < 0.3, the activity was switched off, with the effect turning positive (growth-enhancing). In LB medium, GMs demonstrated a growth promoting effect when the SOC > 0.3 and switched to antibacterial when the SOC < 0.3.

### Intrinsic Oxidative Potential of GMs Is Not the Main Factor Leading to Their Distinct Antibacterial Activity

**Figure 2:**
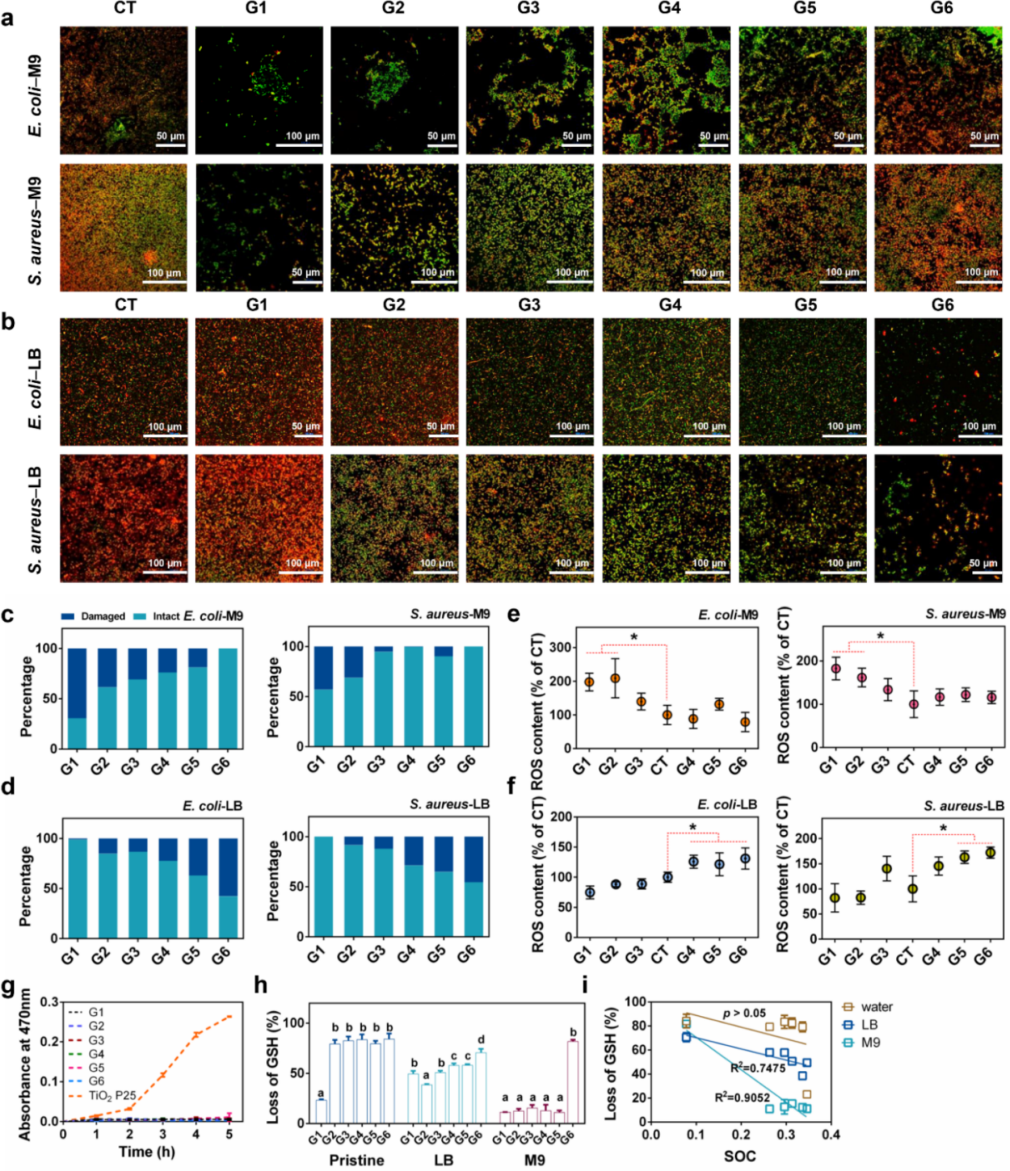
Membrane damage and ROS accumulation in bacterial cells and oxidative potential of GMs. (a, b) Fluorescence images of JC-1 labeled bacterial cells viewed by confocal microscopy, after 48 h incubation with GMs in M9 medium (a) or LB medium (b). Intact membrane was stained with red while damaged membrane was stained with green. (c, d) Percentage of intact/damaged bacterial cells over the control after exposure to GMs in M9 (c) or LB medium (d). (e, f) Relative ROS content in bacterial cells after incubation with GMs in M9 (e) or LB medium (f) for 48 h. * *p* < 0.05 indicates significant difference compared with control (CT) (*n* = 6, mean ± s.d.). (g) Production of superoxide radical anion (O_2_^•–^) by GMs measured by the XTT method. Production of O_2_^•–^ by TiO_2_ nanoparticles under UV radiation was used as a positive control. (h) Percentage of GSH loss due to oxidation by GMs that were preincubated in different media (deionized water, LB, M9 medium) for 3 h. Different lowercase letters indicate significant difference (*n* = 6, mean ± s.d.). (i) correlation of SOC with GSH oxidation capacity of GMs in water, LB or M9 medium (*n* = 6, mean ± s.d.).

To explore the mechanism for the action of the SOC switch, we first examined the membrane integrity of bacteria following exposure to the GMs using the JC-1 staining assay (Figure 2a,b). G1 with high SOC caused serious membrane damage (green color in Figure 2a) in M9 medium as manifested by the high fraction of damaged cells (Figure 2c). The dominant color shifts gradually from green to red with the decrease of SOC, suggesting that the membrane damage was reduced. However, in LB medium, an opposite trend of the color shift and an increased portion of damaged cells was observed with the decrease of SOC (Figure S2b,d).

We found that the ROS content in both bacteria was increased after exposure to G1 and G2 in M9 medium (Figure 2e) and G4, G5, and G6 in LB medium (Figure 2f). Moreover, the percentage of loss of cell viability was negatively correlated with the ROS accumulation in cells (Figure S11), suggesting the occurrence of oxidative damage in bacteria.

Next, we explored the origin of the excessive intracellular ROS accumulation. We first examined whether the GMs *per se* can generate ROS, by measuring the O_2_^•–^ concentrations in the GM suspensions.^2^ Compared to the positive control of a UV-light exposed TiO_2_ suspension, ROS production by the GMs was not detectable (Figure 2g), thus excluding the possibility of antibacterial effects caused by direct generation of ROS from GMs.

Then, we examined another possibility, that GMs may have intrinsic oxidative potential which can oxidize intracellular antioxidants (e.g., glutathione (GSH)), thereby reducing the antioxidative capacity of the bacterial cells.^17^ Since a significant impact of medium composition on the antibacterial activity of GMs was observed as shown above, GMs were preincubated with M9 or LB medium for 3 h before incubation with GSH as comparison. As shown in Figure 2h, the amount of GSH oxidation upon exposure to pristine G2–G6 was much more pronounced than with exposure to G1. This agrees with previous studies that the GSH oxidation capacity of rGO was higher than GO.^2,18^ rGO has a much higher electrical conductivity than GO and, therefore, is more favorable for GSH oxidation, through acting as a bridge to enhance the movement of electrons from GSH to the external environment.^2,19^ However, recent few studies showed opposite results, whereby thermally annealed rGO showed lower GSH oxidative potential than pristine GO.^20,21^ This may be due to the distinct properties of the materials used. The GO used in these studies had a porous structure which distinguishes it from the plate structure of rGO, and the thermal annealing process caused stacking of the sheets and significantly increased the thickness of the rGO which reduced the effective surface that can react with GSH. These changes in the physiochemical properties may have led to the different trend of GSH oxidative potential and antibacterial performance from that observed in our and other studies.

When preincubated with the culture medium, GMs generally showed a significant decrease in their oxidative potential compared to pristine GMs (Figure 2h). *In vitro* experiments and computational modeling have suggested that epoxides and the nearby C–OH groups at the GO surface provide key sites for reaction with GSH,^21^ indicating the important role of exposed GO surface in GSH oxidation. When encountering the culture medium, the basal surface of GMs will be covered by the medium components, which may alter their surface properties (which will be discussed in the following sections) and subsequently change their oxidative potential. We also found that the percentage of loss of GSH after incubation with preincubated GMs was negatively correlated with the SOC (Figure 2i). This cannot explain the positive effects of G1 and G2 and insignificant effects of G3 and G4 on the bacterial growth in LB medium, as they have strong oxidative potential which should have caused antibacterial effects. Likewise, although the GSH oxidation may contribute to the antibacterial activity of G1 and G2 in M9 medium, the overall patterns of the oxidative potential are not in accordance with their antibacterial activity. Linear regression analysis further showed that the oxidative potential of GMs was not linearly correlated with their antibacterial activity (Figure S12). Taken together, all these findings suggest that chemical oxidation of intracellular antioxidants is unlikely the main contributor to the excessive intracellular ROS accumulation and the antibacterial activity of GMs. Instead, physical interaction may play a significant role which will be explored next. If a physical damage occurs, then the cell will produce ROS as a defending response. However, when the ROS is excessive, cell death will occur.

### SOC-Dependent Physical Disruption of Bacterial Membrane Is the Main Contributor to the Antibacterial Activity

**Figure 3:**
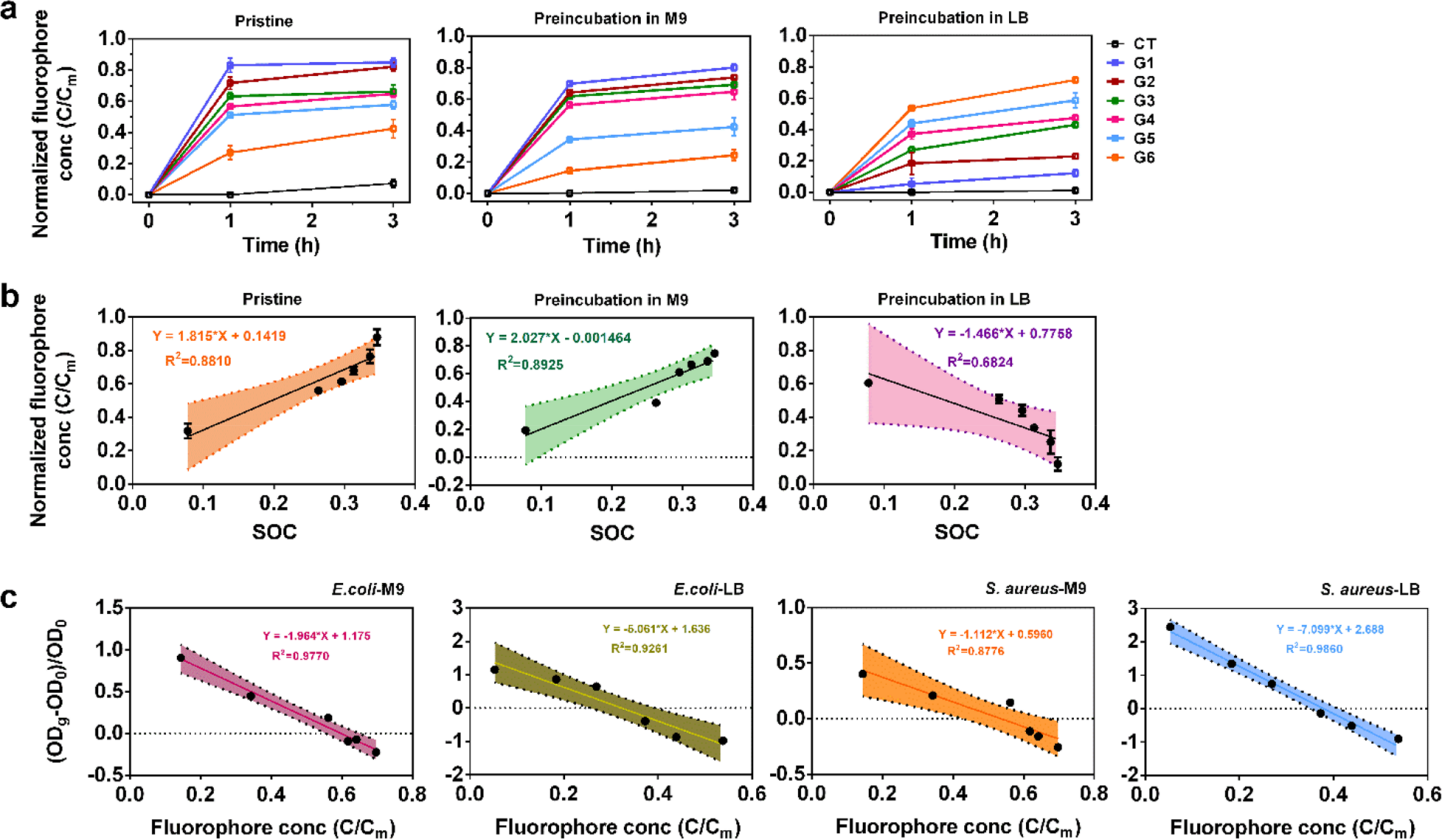
SOC-dependent physical disruption of bacterial cell membrane contributed to the differential antibacterial activity of GMs. (a) Time-dependent leakage of fluorophore from liposome vesicles after treatment with 100 mg/L of pristine GMs as well as GMs preincubated in M9 or LB media for 3 h. *C*/*C*_m_, the normalized fraction of the leaked fluorophore, indicates the loss of membrane integrity due to damage to the lipid membrane. (b) SOC was positively correlated with the fluorophore leakage from the lipid vesicles (at 1 h) caused by pristine GMs and GMs preincubated with M9, but negatively correlated with the fluorophore leakage from the lipid vesicles caused by GMs preincubated with LB. (c) The fraction of the leaked fluorophore (at 1 h) caused by the GMs was positively correlated with the percent of GM-induced loss of cell viability (OD_600_) at 48 h.

In order to evaluate the physical interactions, we used liposome vesicles (Figure S13) encapsulating a fluorescent dye as a model membrane.^13^ This model allows quantification of the membrane damage by measuring the leakage of fluorophore into the extravesicular solution. All the pristine GMs caused significant leakage of fluorophore, and the leakage increased with increasing incubation time. The highest leakage was observed for G1, and the leakage decreased gradually from G1 to G6 (Figure 3a). This demonstrated that physical interaction alone could cause lipid membrane damage, and that the damage was SOC-dependent.

Considering the strong effects of culture medium on the antibacterial affects and the intrinsic oxidative potential of GMs, we also examined the impacts of culture medium on the physical action. GMs were first preincubated with M9 or LB medium for 3 h before incubation with the liposome. Compared to pristine GMs, preincubation with M9 medium reduced the membrane damage as shown by the reduced fluorophore leakage (Figure 3a), although the trend of membrane damage follows the same order. However, preincubation with LB led to a reversed pattern of membrane damage, which decreased with increasing SOC (Figure 3a). Further linear regression analysis showed that the leakage of fluorophore at 1 h (Figure 3b) and 3 h (Figure S14) was positively correlated with the SOC for either pristine (*R*^2^ = 0.8810) or M9 preincubated (*R*^2^ = 0.8925) GMs but negatively correlated with LB preincubated GMs (*R*^2^ = 0.6824). The patterns of the membrane damage caused by GMs were correlated very well with their antibacterial activity (*R*^2^ > 0.8776) (Figures 3c, S15, and S16). This demonstrated that physical disruption contributed significantly to the differential antibacterial activity of GMs with different SOC.

The medium content has been suggested to affect the aggregation states of GMs thereby affecting their physical interaction with bacteria as well as the antibacterial performance.^22^ So, next, we examined the stability of the GMs in M9 and LB media by examining their zeta potentials and hydrodynamic sizes over 48 h. In M9 medium, the zeta potentials of GMs were slightly different at the beginning (0 h); however, the difference between the GMs diminished after 48 h (Figure S17). In LB medium, the differences among the GMs were not profound at either time point. The hydrodynamic size of the GMs with different SOC differed at the beginning (0 h), with GMs of high SOC showing smaller size (Figure S18). However, the subsequent aggregation occurred very quickly (i.e., within 10 min), with the size increasing significantly for all GMs, and the difference between the GMs diminishing and disappearing, and the sizes remained constant even after 24 h. Since our antibacterial experiments were performed over 48 h, (lack of) stability was not a factor contributing to the different antibacterial results.

### Transition of Parallel to Perpendicular Interaction Mode Controlled by an SOC Threshold

Given the critical role of SOC and the effect of the culture medium on the GMs-induced physical damage of the phospholipid membranes, we further investigated the underlying mechanisms. Until now, the interaction mode of GO with cell membranes was mostly evidenced by computational simulations, with only limited experimental studies having observed the interaction of GMs with bacterial membranes using SEM. In a study performed by Guo et al., SEM imaging showed that GO wrapped or covered the bacteria.^23^ Perpendicular penetration of animal cells was observed in another study for pristine graphene,^11^ and this is the only study that visually observed the penetration of GMs into the cell membrane.

**Figure 4:**
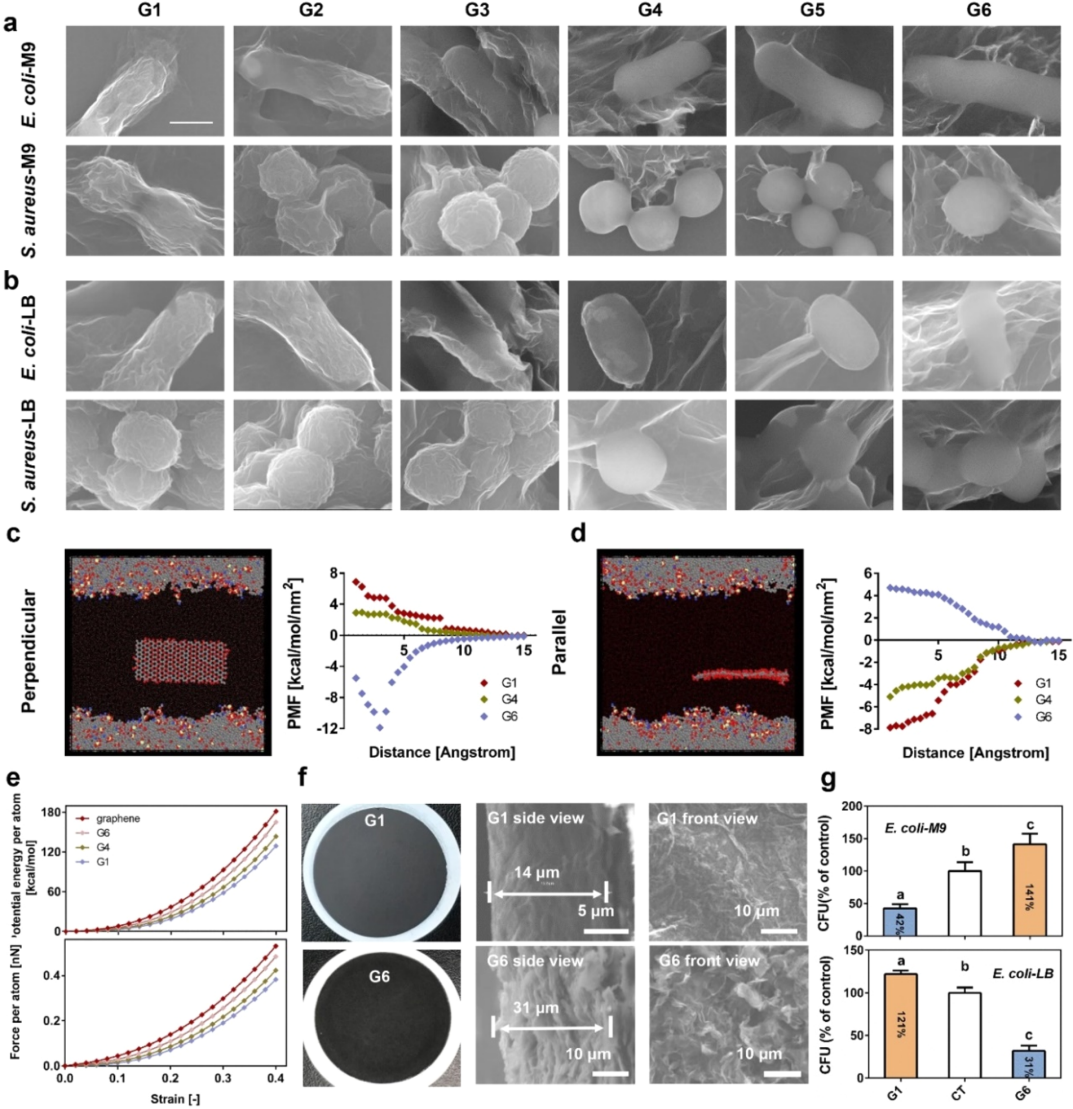
Interaction modes of GMs with different SOC with bacteria. (a, b) SEM images showing that G1, G2, and G3 wrap around the bacterial in M9 (a) or LB (b) medium. G4, G5, and G6 contact bacterial cells with their lateral edge in M9 (a) or LB (b) medium. (c, d) Representative configurations of GM nanosheet aligned perpendicular or parallel to the surface of the lipid membrane during the constrained MD simulations. For clarity, the hydrogen atoms are hidden while the oxygen atoms of all water molecules have a reduced size. Gray/red/white/blue/green colors were used for the carbon/oxygen/hydrogen/nitrogen/phosphorus atoms in the system. PMF was plotted as a function of the distance of the GMs nanosheet to the surface of the lipid membrane. (e) The rigidity of the GMs was derived based on the variation of the potential energy per atom and force per atom of GMs. High *y*-axis value indicates high rigidity. (f) Photos and SEM images of G1 and G6 films showing G1 film with a relatively smooth surface while G6 film with sharp edges. (g) Relative numbers of viable *E. coli* colonies formed after 3 h of incubation with G1 or G6 film in M9 or LB medium (*n* = 6, mean ± s.d.). Different lowercase letters indicate significant differences (*n* = 6, mean ± s.d.).

From these limited examples, it seems that GO and graphene have different interaction modes with cell membrane. One may deduce that GMs with different SOC may have different interaction modes with bacteria given the SOC difference between GO and graphene. Using SEM, we observed a distinct transition of the interaction mode from parallel to perpendicular with decreasing SOC, regardless of the culture medium (Figures 4a,b and S19). G1, G2, and G3 with relatively high SOC (>0.3) exclusively wrapped the bacterial cells, making a wrinkled surface (Figures 4a,b and S20), which suggests a strong interaction between the graphene sheets and the cell surface. We also found occasionally that although some fragments of G1–G3 cannot wrap bacteria due to their small size, they closely attached onto the cell surface (Figure S21a,b), suggesting that GMs with high SOC exclusively attach in parallel to the bacteria surface. On the other hand, G4, G5, and G6 attached to the cells by their edges, suggesting a perpendicular interaction as proposed in many computational simulations. We also found that some bacteria were randomly covered by G4–G6 but only loosely with no wrinkle morphology evident (Figure S21c,d). These findings provide experimental evidence that the interaction of GMs with bacterial cells cannot be simply described by a single mode; rather, it varied between parallel attachment and edge contact, which is well controlled by an SOC threshold.

The transition of the interaction mode with the variation of SOC of GMs was further confirmed by MD simulations of the interaction of G1, G4 and G6 (Figure S22) with a model cell membrane (palmitoyloleoylphosphatidylethanolamine, POPE) (details are given in the Experimental Methods section and the SI).

GMs orientation relative to the membrane surface was fixed to be either perpendicular (Figure 4c) or parallel (Figure 4d). The distance between the center-of-mass of the membrane and either the closest GM edge (perpendicular orientation) or the GM center-of-mass (parallel orientation) was varied, and the total force exerted on them (i.e., the potential of mean force (PMF) between GMs and the membrane) was quantified. Positive values in the PMF signify repulsive interactions between the two objects leading to their separation while negative values correspond to attractive interactions resulting in their aggregation. The profiles presented in Figure 4c,d verify that nanosheets approaching the cell membrane perpendicularly are thermodynamically favored when they have a low SOC (G6 in Figure 4c). If the nanosheets have high SOC, then a parallel orientation is preferred, as shown by the negative PMF (Figure 4d). The PMF profiles also suggest that the membrane–GMs interaction is a thermodynamically spontaneous process as shown by the increased absolute PMF values as the GMs approach the membrane. The interaction of pure graphene with POPE membrane was also simulated (Figure S23). The negative PMF at a perpendicular orientation suggests that there is an attractive force between graphene and the lipid membrane. However, a strong repelling force (positive PMF) was observed when the orientation of the graphene sheet is parallel to the membrane.

The mechanism underlying the SOC-dependent orientation of GMs on lipid membranes was further explored by examining the sheet stiffness. Computational modeling suggested that the rigidity of GMs decreased with increasing SOC (Figure 4e). Graphene (without surface oxygen) showed the highest rigidity. The results agree with the previous experimental findings that rGO is much more rigid than GO. This is due to the fact that introducing surface functional groups breaks the plane lattice and thus increases the interlayer thickness, which reduces the stiffness.^24^ The stiffness of graphene as well as rGO allows them to stand freely, while the super flexibility and bendability of GO facilitates folding and wrapping.^25^ This difference in mechanical properties is thus an important character that might lead to the distinct interaction modes of GMs of different SOC with bacteria.

Taken together, these results clearly demonstrate that GO damages bacteria by parallel interaction rather than by cutting the bacterial cell membrane as parallel attachment is preferable in the suspension phase, while reduced GO (SOC < 0.3) damages bacteria with their edges. Neither interaction mode necessarily kills bacteria (Figure 1). Rather, the overall impact depends also on the medium composition. GO only showed antibacterial activity in M9 medium, while rGO was antibacterial only in LB medium.

This mechanism was further confirmed by studying the antibacterial activity of G1 and G6 films made by vacuum filtration. G1 with a relatively smooth surface (Figure 4f) showed strong antibacterial effects on *E. coli* in M9 medium (Figure 4g). This demonstrated that a parallel interaction between the basal plane of G1 and the bacterial cell membrane contributes to the bacterial death. This also indirectly demonstrated that trapping-induced nutrient deficiency is not the main (at least not the only) mechanism under a wrapping mode in the suspension assay, because in the film experiments there was no chance for G1 to wrap/trap the bacteria, but it still killed the bacteria. In contrast, the G6 film with sharp edges (Figure 4f) showed bactericidal effects only in LB medium but exerted beneficial effects in M9 medium, further demonstrating that perpendicular interaction also does not necessarily kill bacteria. The film experiments also demonstrated that particle (sheet) stability is not the main factor leading to the SOC-dependent interaction mode and antibacterial activity, because the GO or rGO were present as a rigid and immobile film on poly(ether sulfone) membranes and thus their aggregation/agglomeration is impossible.

### SOC-Dependent Protein Corona Formation on GMs Changes Their Antibacterial Activity

**Figure 5:**
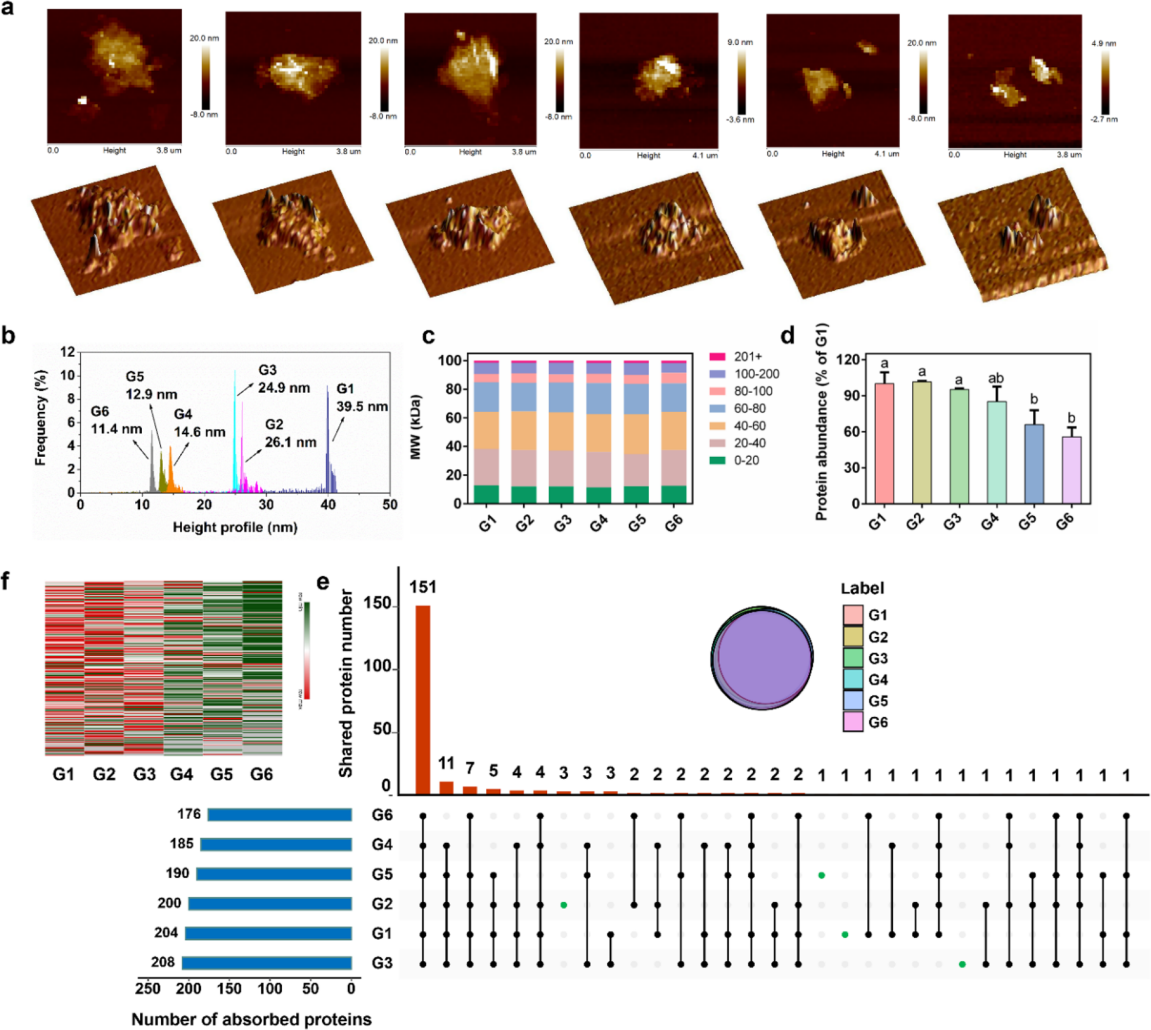
Corona formation on the surface of GMs. (a) AFM images of GMs after incubation in LB media for 3 h. (b) Thickness of GMs. (c) Classification of all proteins identified in the corona of GMs according to their molecular weight. (d) Total abundance of proteins absorbed on the surface of the GMs (*n* = 3, mean ± s.d.). (e) UpSet plot of the intersection of absorbed proteins in the coronas of the different GMs. The left bar figure shows the number of absorbed proteins for different GMs. The inner figure is a Venn figure showing the highly overlapped number of proteins. (f) Heatmap of all proteins identified in the corona of GMs showing GMs with high SOC adsorb more proteins than those with low SOC. A complete list of the absorbed proteins is included in Table S4.

Given the critical role of culture medium in determining the antibacterial effects of GMs, we next explored the underlying mechanism. M9 medium, also known as minimal medium, provides only basal salts and a small fraction of carbon source (e.g., sucrose) to maintain the bacterial growth. Results in this medium should roughly reflect the intrinsic antibacterial activity of the pristine GMs. However, in LB medium, which is a nutrient-rich medium, proteins and other bioactive molecules from the culture media may be absorbed onto the GMs, forming a “corona” and changing the surface chemistry of the GMs.^26^ The adsorption process generally takes place more quickly than material–cell interactions.^27^ We hypothesize that corona formation may be responsible for the reversed antibacterial effects in LB medium compared to M9 medium. To verify this hypothesis, we first measured the thickness of the GM surface after 3 h of incubation in LB and M9 media using AFM. M9 incubation did not significantly change the thickness of all GMs compared to pristine materials (∼1 nm) as expected (Figure S24). However, the thickness of the GMs increased significantly when incubated in LB medium, and the thickness increased gradually with the increase of SOC (Figure 5a,b), suggesting an SOC-dependent corona formation.

Given the protein based organic composition of the LB medium, adsorbates on the GM surfaces after 3 h of incubation with LB medium were then subject to LC-MS/MS for proteomic analysis. The results showed that the adsorbed proteins cover all MW ranges, with proteins of <80 kDa accounting for more than 60% of the total adsorbed corona (Figure 5c). The total abundance of proteins that were adsorbed on the GM surfaces decreased with decreasing SOC (Figure 5d), which is consistent with the AFM results. Although there was a considerable number of overlapping proteins (151 out of 221) among the six GMs (Figure 5e), the abundance of each protein on the GM surfaces was significantly different between the different GMs as shown in the heatmap in Figure 5f. The GMs with high SOC actually acted as a platform to enrich the proteins as well as other nutrients to support bacterial growth when wrapping the bacterial surface, which was also observed in a previous study.^23^ However, most of the defects of reduced GO (i.e., G4–G6 in this case) are at their ends;^28^ thus, the defects at the basal plane which are critical sites for interacting with proteins are fewer than those of the more oxidized GO (G1–G3), which supports the distinct decrease in adsorption capability observed and its correlation with SOC.

In order to demonstrate the importance of the SOC concept in real world applications, we also measured the antibacterial activity of GMs in simulated body fluid (SBF) and simulated wastewater (SWW). SBF contains only salts, at concentrations similar to those in human blood plasma, while the SWW simulated municipal wastewater containing large amounts of organic nutrients and salts. Consistently, SOC-induced switching of the antibacterial performance in SBF and SWW media were similar to those presented above in M9 and LB media, respectively (Figures S25–S28), further demonstrating the SOC concept and the critical role of the biomolecule corona.

## Conclusions

**Figure 6:**
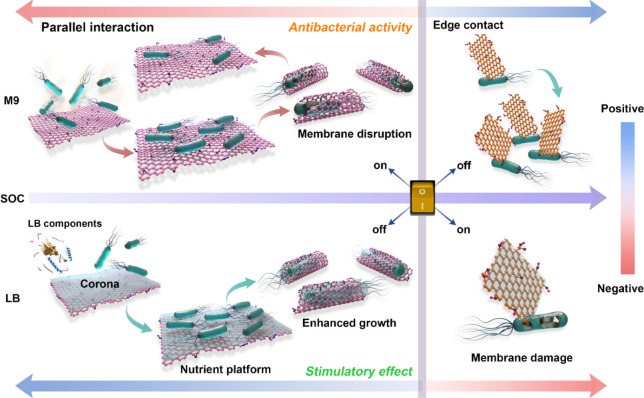
Schematic illustration of the SOC-threshold induced switch of interaction mode and the antibacterial activity of GMs.

In summary, we found that variation of the SOC of GMs is a key factor leading to the apparent uncertainties on the physical mechanism(s) of the antibacterial activity of GMs in literature. A SOC threshold was identified, before and after which the GMs showed distinct antibacterial effects based on a switch in the mode of physical interaction (Figure 6). We demonstrated that physical disruption was the major contributor to the antibacterial activity, with high SOC favoring parallel wrapping and low SOC favoring perpendicular contact. Protein corona formation on GMs deactivated the antibacterial effects of GMs with high SOC and acted as platform for bacterial growth. It should be noted that the SOC-induced switching threshold can vary depending on other intrinsic parameters such as lateral size and thickness, which needs to be considered in subsequent studies; thus, SOC and switching threshold need to be determined explicitly for the specific GMs tested. This study highlights that the SOC switch can be used as a criterion for evaluating the antibacterial effects of GMs and can feed into computational modeling for safe-by-design of modified GMs. The SOC switch can also potentially be used as a design criterion for sensing and delivery applications of GMs. The study also provides direct evidence that SOC should be considered as a determining property for the definition/classification of GMs in the context of human health and environmental safety.

## Experimental Methods

### Antibacterial Activity of GMs

*E. coli* (K-12) and *S. aureus* (23656) cells in the midexponential growth phase were used for all experiments. 100 mg/L GM suspensions in different medium were chosen based on our preliminary experiments. The *E. coli* and *S. aureus* were exposed to GM suspensions for 48 h, and the total cell growth (OD_600_) and biofilm formation (OD_540_) were then analyzed as described in our previous study.^23^ Cell viability was also determined using LIVE/DEAD BacLight Bacterial Viability Kit and the plate-counting colony formation assay. The antibacterial activity of G1 and G6 film, produced by a vacuum filtration method as described in the SI, was also evaluated. *E. coli* were exposed to G1 and G6 film in different media for 3 h at room temperature and the plate-counting colony forming assay was then performed. See details in the SI.

### Examination of Membrane Integrity

Bacterial membrane integrity was measured using a JC-1 assay kit (Beyotime Biotech, Nantong, China) as per the manufacturer’s protocol. JC-1 is a lipophilic cationic dye that exhibits a spectral shift from red to green as membrane potential decreases and thus can be used to assess membrane integrity. See full details in the SI.

### Measurement of ROS in Bacterial Cells

ROS levels in *E. coli* and *S. aureus* cells under different treatments were detected using 2′,7′-dichlorodihydrofluorescein diacetate (DCFH-DA) followed the procedure of Simon-Deckers et al.^29^ with minor modification. See the SI for details.

### Measurement of O_2_^•–^ in GM Suspensions

The generation of O_2_^•–^ was assessed in a solution of dispersed GMs using 2,3-bis(2-methoxy-4-nitro-5-sulfophenyl)-2*H*-tetrazolium-5-carboxanilide (XTT; Sigma-Aldrich) as a probe. XTT reacts with O_2_^•–^ to generate XTT-formazan. Solutions containing 100 μg/mL GM or UV-light exposed TiO_2_ suspension as positive control and 100 μM XTT were stored in the dark at room temperature. Aliquots of the solution were taken at various time points for kinetic analysis of O_2_^•–^ generation. Sample (1 mL) was added into a cuvette and the formation of XTT-formazan was determined via absorption at 470 nm using a UV–vis spectrophotometer (RF-5301PC; Shimadzu).

### GSH Oxidation Assay

GSH oxidation mediated by the GMs was measured according to the approach described previously with a minor modification.^3^ Briefly, GMs were incubated in LB or M9 medium for 3 h. Then, pristine GMs, GMs in LB or in M9 medium (100 mg/L) were added into a 50 mL Erlenmeyer flask containing 20 mL of 50 mM bicarbonate buffer (pH 8.6) to react at room temperature in the dark, with constant agitation for 3 h. The amount of nonoxidized GSH was quantified spectrophotometrically using Ellman’s reagent (5,5′-dithiobis (2-nitrobenzoic acid), DTNB), which can react with thiol groups of GSH to yield 3-thio-6-nitrobenzoate (TNB). The reaction medium was filtered through 0.22 μm syringe filters (Millex GP Filter Unit, Coringhwohill, County Cork, Ireland). Then, 900 μL of the filtrates was added to 1.57 mL of Tris-HCl buffer (pH 8.3), followed by addition of 30 μL of 100 mM DTNB solution. The amount of thiol remaining in the reaction medium was quantified by measuring the absorbance at 412 nm.

### Physical Interaction of GMs with Liposomes

Dye-leakage experiments were performed by exposing liposomes encapsulating a fluorescent dye solution [50 mM 3-(*N*-morpholino) propanesulfonic acid (MOPS), 50 mM 5(6) carboxyfluorescein (pH 7.5)] to GM suspensions according to previously reported methods.^13^

### SEM Images

After treatment with GM suspension for 48 h, *E. coli* and *S. aureus* cells were collected by centrifugation at 8000 rpm for 10 min and fixed with 2.5% glutaraldehyde overnight at 4 °C. Cells were then dehydrated in graded ethanol solutions (30%, 50%, 70%, 90% once, and 100% twice) and resuspended in ethyl alcohol absolute. The cell suspensions were dropped onto the silicon glide, freeze-dried, and observed by SEM (S-4800, Hitachi, Japan).^13^

### Thickness Analysis by AFM

Suspensions of GMs in M9 or LB medium (100 mg/L) were prepared and sonicated at 200 W for 10 min. The suspensions were then incubated at 37 °C in darkness for 4 h. The suspensions were then centrifuged at 12000 rpm for 30 min, and the pellets were rinsed with deionized water 3 times. The final pellets were resuspended in deionized water, and 20 μL of suspension was dropped onto a mica wafer for AFM characterization.

### Protein Corona Composition Analyzed by Proteomic Analysis

100 μL of 1 mg/mL GM suspension was incubated with 500 μL of LB medium for 3 h at 37 °C, shaking at 300 rpm. Then samples were collected for protein corona analysis as described previously,^30^ with detailed sample processing and analysis as described in the SI. The MS and MS/MS scans were searched against the Uniprot database (download version 08, Dec. 2020, 257613 sequence entries) and the *Saccharomyces cerevisiae* database (download version 10, Sept. 2020, 5983 sequence entries) using Proteome Discoverer 2.1 (ThermoFisher Scientific). Fixed modification was carbamidomethyl-cysteine; variable modifications were oxidation of methionine and acetylation of the protein N-terminus. The precursor mass tolerance was 10 ppm, and the MS/MS mass tolerance was 0.02 Da. Two missed cleavages were allowed and identified proteins are accepted as a real hit protein with at least two high confidence peptides. The mass spectrometry proteomics data have been deposited to the ProteomeXchange Consortium via the PRIDE partner repository with the data set identifier PXD027909.

### Molecular Dynamics (MD) Simulation

MD simulations of graphene, GO, and reduced GO nanosheets (G1, G4, and G6) interacting with a model *E. coli* outer membrane in an aqueous environment were performed. Due to the complexity of the membrane structure, palmitoyloleoylphosphatidylethanolamine (POPE) lipids were used since they are one of the most abundantly found in Gram-negative bacteria. This selection is in line with a previous study.^11^ Detailed simulation parameters are described in the SI. The rigidity of GMs was also gained by carrying out tensile loading simulations to the GMs as described in the SI.

### Statistical Analysis

All tests were repeated at least three times (with many *n* = 6). Data were expressed as mean ± SD (standard deviation). Statistical significance was analyzed by ANOVA or student’s *t* test. *p* < 0.05 was considered as the level of significance.

## Data Availability

The data supporting the findings of this study are available within the paper and its Supporting Information and in the PRIDE repository with the identifier PXD027909. Source data are provided with this paper.

## Supporting Information Available

Details materials and methods, survey of the SOC literature of GMs on the antibacterial effects, the characterization of GMs, the antibacterial effects and the potential antibacterial mechanism of GMs, liposome leakage, and MD simulation studies (PDF)
