# Supplementary material for: Defining the Surface Oxygen Threshold That Switches the Interaction Mode of Graphene Oxide with Bacteria
Source: ACS Nano. 2023 Feb 26;17(7):6350–61. doi: 10.1021/acsnano.2c10961 (PMC10100553; doi:10.1021/acsnano.2c10961)
Supplement: Supplementary file 1 — nn2c10961_si_001.pdf [file nn2c10961_si_001.pdf]

Supplementary Information

Defining the surface oxygen threshold that switches the interaction mode of graphene oxide with bacteria

Zhiling Guo, [*⊥,†] *Peng Zhang,* [*⊥,†,‡] *Changjian Xie,* [*⊥,ƻ] *Evangelos Voyiatzis* [§], *Klaus Faserl* [∥], *Andrew J Chetwynd* [†], *Fazel Abdolahpur Monikh* [¶], *Georgia Melagraki* [§], *Zhiyong Zhang* [τ], *Willie J.G.M. Peijnenburg* [∮], *Antreas Afantitis* [§], *Chunying Chen* [≌], *and Iseult Lynch* [†]

[†] *School of Geography, Earth and Environmental Sciences, University of Birmingham, Edgbaston, Birmingham B15 2TT*
[‡] *Department of Environmental Science and Engineering, University of Science and Technology of China, Hefei 230026, China*
[ƻ] *School of life Sciences and medicine, Shandong University of Technology, Zibo 255000, Shandong, China*
[§] *Nanoinformatics Department, NovaMechanics Ltd., Nicosia, 1065 Cyprus*
[∥] *Institute of Medical Biochemistry, Medical University of Innsbruck, 6020 Innsbruck, Austria*
[¶] *Department of Environmental & Biological Sciences, University of Eastern Finland, P.O. Box 111, Joensuu, FI-80101, Finland*
[τ] *Key Laboratory for Biological Effects of Nanomaterials and Nanosafety, Institute of High Energy Physics, Chinese Academy of Sciences, Beijing 100049, China; School of Nuclear Science and Technology, University of Chinese Academy of Sciences, Beijing 100049, China*
[∮] *Institute of Environmental Sciences (CML), Leiden University, Einsteinweg 2, 2333 CC Leiden, the Netherlands; National Institute of Public Health and the Environment (RIVM), Center for Safety of Substances and Products, Bilthoven, the Netherlands*
[≌] *CAS Center for Excellence in Nanoscience and CAS Key Laboratory for Biomedical Effects of Nanomaterials and Nanosafety, National Center for Nanoscience and Technology of China, Beijing 100190, China; Research Unit of Nanoscience and Technology, Chinese Academy of Medical Sciences, Beijing 100039, China; GBA National Institute for Nanotechnology Innovation, Guangdong 510700, China; Research Unit of Nanoscience and Technology, Chinese Academy of Medical Sciences, Beijing 100021, China*

Corresponding Authors

* *Email:* z.guo@bham.ac.uk (Z. Guo); p.zhang.1@bham.ac.uk (P. Zhang); xiecj@sdut.edu.cn (C. Xie).

Methods

Preparation of GMs

The GO (G1) was synthesized by chemical exfoliation of graphite oxide obtained by classic Hummers method. [1] The as-received graphite oxide was filtered and washed with HCl solution (*v:v*, 1:10) followed by dialysis (MW cut off: 8000) for one week to remove metal residues prior to the synthesis. The washed graphite oxide was then dispersed in deionized water and sonicated for 30 min to obtain GO. G6 with the lowest surface oxygen content (SOC) was produced by oxidizing graphene with a H_2SO_4 / HNO_3 mixture at room temperature, as described by Majeed *et al.* [2] G2, G3, G4 and G5 were prepared by reducing G1 with ascorbic acid, a mild reducing agent, for 6 h (G2), 12 h (G3), 24 h (G4), and 48 h (G5). [3] The obtained GMs were further purified by dialysis for one week to remove any residual chemicals. G1 and G6 films were made by a vacuum filtration method. [4] In brief, 1 mg/mL G1 and G6 water suspensions were filtered through a PVDF membrane (47 mm in diameter, 0.22 μm pore size) via vacuum at room temperature.

Characterization of GMs

The chemical composition of the GMs was analysed by XPS (ESCALAB 250Xi, Thermo Scientific, USA). UV-vis spectra of GM suspensions (100 mg/L) were recorded using a UV-vis spectrophotometer (UV-2700, Shimadzu, Japan). The surface morphology of the G1 and G6 films was characterized using a Dimension FastScan AFM system (Multimode 8, Bruker, USA). The lateral size and thickness of the GMs was analyzed using NanoScope (Bruker, USA). The hydrodynamic size and zeta potential of the GMs in M9 and LB medium were measured by Zetasizer (Nano ZS, Malvern Panalytical, UK)

Antibacterial activity test of GMs in LB/M9/SBF/SSW medium

In addition to the antibacterial activity test of GMs in LB and M9 media, we also explored whether the SOC dependent effects and the SOC switch exist in more realistic environmental and biological conditions, for which we used simulated body fluid (SBF) and simulated wastewater (SWW) as

representatives. The SBF and SWW were prepared following the procedures described by Kokubo *et al.,*[5] and Taştan *et al.,* [6] respectively. The use of SBF is to simulate a scenario in human body fluid in which GMs might be used for treatment of bacterial infection. The concentrations of salts in SBF are the same as those in human blood plasma. The SBF (pH 7.4) contains 142 mM Na^+, 5 mM K^+, 1.5 mM Mg^{2+}, 2.5 mM Ca^{2+}, 103 mM Cl^-, 27 mM HCO_3^-, 1 mM HPO_4^{2-} and 0.5 mM SO_4^{2-}. The use of SWW is to simulate the scenario whereby GMs are used for water treatment. The ingredients of SWW are 25 mg/L peptone, 80 mg/L yeast extract, 200 mg/L starch, 35 mg/L sunflower oil, 150 mg/L ammonium acetate, 26 mg/L KH_2PO_4, 6 mg/L $MgHPO_4 \cdot 3H_2O$, 2000 mg/L glucose, 26 mg/L K_2HPO_4, 50 mg/L urea, 8 mg/L $FeSO_4 \cdot 7H_2O$, and 160 mg/L whey powder. The SBF is similar to M9 medium, most of which is salts, while SWW is similar to LB, which is a nutrient rich media.

The *E. coli* (K12) and *S. aureus* (23656) were purchased from the Institute of Microbiology, Chinese Academy of Sciences. Bacteria were grown on Luria-Bertani (LB) agar plates at 37 ^oC for 24 h. A single colony was picked and inoculated in LB medium and incubated overnight at 37 ^oC under 220 rpm shaking speed. The cultures were harvested in the mid-exponential growth phase by centrifugation at 6000 rpm for 10 min and washed with 0.9% NaCl solution three times. The pellets were resuspended in LB to obtain a final bacterial suspension with $OD_{600} = 0.5$ for all the following experiments.

GM suspensions (100 mg/L) of 300 μL were pipetted into sterilized 96-well flat-bottom microtiter plates. 3 μL *E. coli* and *S. aureus* suspensions were inoculated into the wells. Wells with GM suspensions but without bacteria, and LB/M9/SBF/SSW medium without bacteria were set as bacteria-free blanks. LB/M9/SBF/SSW medium with bacteria were set as the untreated controls. Each treatment has six replicates. All plates were incubated statically at 37 ^oC for 48 h in the dark. Total cell (cells in the suspensions and the cell aggregates on the bottom) growth was measured by recording the OD_{600} with a microplate reader (Infinite 200 Pro, Tecan, Switzerland). The readings from wells without bacteria were averaged out and subtracted from the readings from wells with bacteria.

Biofilm mass that was formed on the bottom of the well was quantified by a crystal violet staining method as described in previous literature. [7] Briefly, the suspensions in the wells were discarded after measurement of the total cell growth. The wells were then rinsed with PBS buffer (10 mM, pH 7.4) three

times to remove the suspended and unbound cells. Crystal violet solution (250 μL 0.1%) was added into each well and incubated at room temperature for 20 min, which allowed staining of the biofilm. The solutions were then discarded and the dye in the stained biofilm were released by adding 300 μL acetone/ethanol (1:4, *v/v*), and the absorbance at 540 nm (OD_{540}) was recorded under a microplate reader. Readings from blanks were averaged out and subtracted from that with bacteria.

Biofilm formation was also determined using a LIVE/DEAD BacLight Bacterial Viability Kit. Briefly, bacteria suspension (10^8 CFU/mL) were seeded in 35 mm confocal dishes containing GM suspensions (100 mg/L) in LB or M9 medium. After incubation for 48 h, the suspensions in the dishes were removed and the dishes were rinsed with PBS buffer (10 mM, pH 7.4) three times to remove the suspended and unbound cells. Then remaining biofilm were stained with the LIVE/DEAD BacLight Bacterial Viability Kit (Invitrogen Corp., Carlsbad, CA) according to the provided protocol. Images of the biofilm were taken on a confocal laser scanning microscopy (CLSM, Nikon A1, Tokyo, Japan).

For the plate-counting colony assay, *E. coli* bacterial cells (10^8 CFU/mL) were firstly incubated with GM suspension in M9 or LB medium for 3 h. At the end of the exposure period, the bacterial suspension was bath-sonicated for 10 min to break aggregates. Then, bacterial suspensions were immediately cultured on LB agar media and incubated overnight at 37 °C for colony forming unit (CFU) enumeration.

For G1 and G6 film exposure experiment, the *E. coli* bacterial suspension (10^8 CFU/mL) were exposed to films in M9 or LB medium for 3 h at room temperature. After discarding the excess bacterial suspension, the film was rinsed with 5 mL saline solution (PBS buffer, 10 mM, pH 7.4) to wash unattached cells from the film. The film was then transferred to 10 mL saline solution and sonicated for 10 min in an ultrasonic bath to detach bacteria from the film surface. The detached bacteria were subsequently cultured on solid LB agar and incubated overnight at 37 °C for CFU enumeration.

Examination of membrane integrity

Bacterial membrane integrity was measured by using a JC-1 assay kit (Beyotime Biotech, Nantong, China) as *per* the manufacturer's protocol. Briefly, bacteria suspensions (10^8 CFU/mL) were seeded in 35 mm confocal dishes containing 100 mg/L GM suspensions in LB or M9 medium, with each treatment in triplicates. After 48 h incubation, the bacterial cells were collected and washed with PBS thoroughly, and

then incubated with 10 μmol/L JC-1 dye for 15 min at 37 °C in the dark followed by washing with JC-1 staining buffer three times. The fluorescence was measured using a microplate reader (Infinite 200 Pro, Tecan, Switzerland) with the filters 490/20 (excitation) and 530/20 (emission) for green and 525/20 (excitation) and 590/35 (emission) for red. The ratio of red and green fluorescence was calculated. The biofilm was then imaged by confocal laser scanning microscopy (CLSM, Nikon A1, Tokyo, Japan) at 488 nm/530 nm excitation/emission wavelength.

Measurement of ROS accumulation in bacteria

ROS levels in *E. coli* and *S. aureus* cells under different treatments were detected using 2', 7'-dichlorodihydrofluorescein diacetate (DCFH-DA). Briefly, 300 μL GM suspension (100 mg/L) in LB or M9 medium were added to the 96-well plates. 3 μL *E. coli* or *S. aureus* suspensions were inoculated into the 96-wells. Wells with GM suspensions but without bacteria, and LB/M9 medium without bacteria were set as bacteria-free blanks. LB and M9 medium with bacteria were set as the untreated controls. Each treatment has six replicates. After 48 h incubation, *E. coli* and *S. aureus* cells were collected and washed with PBS thoroughly and resuspended in PBS with 10 μmol/L DCFH-DA. After 30 min incubation at 37 ℃ in the dark, fluorescence intensities were measured with excitation at 488 nm and emission at 525 nm on a microplate reader (infinite M200 PRO).

Protein corona formation on GMs

100 μL of 1 mg/mL particles were incubated with 500 μL of LB medium for 3 h at 37 °C, shaking at 300 rpm. NMs and their associated corona were centrifuged for 10 min at 5000 g and the protein supernatant (soft corona) was removed. The particles were washed once with 250 μL of PBS buffer and then twice with 250 μL of ammonium bicarbonate buffer (ABC buffer) (100 mM, pH 8.0) (>99.5%, Thermo Scientific) by centrifuging for 10 min at 5000 g and removing the supernatant. NMs were transferred to a fresh vial after the second ABC wash.

On-particle protein digestion

To reduce protein disulfide bonds the NM-corona pellet was dispersed in 20 μL ABC buffer (100 mM, pH 8.0) containing 10 mM dithiothreitol (>99%, Roth, Karlsruche, Germany) and 0.5% (*w/v)* sodium deoxycholate (SDC) followed by incubation for 30 min at 56 °C. For enzymatic digestion 0.5 μg of

trypsin (Sequencing grade, Promega), in 20 µL ABC buffer (containing 0.5% (*w/v*) SDC) was added and the sample was incubated for 16 h at 37 °C. Cysteines were alkylated with 20 µL iodoacetamide (>99%, Sigma), 55 mM in 100 mM ABC buffer, at room temperature for 20 min. SDC was precipitated by addition of formic acid (FA) and the resulting peptides in the supernatant were ZipTip enriched using C18 Tips (100 µL, Pierce, Thermo Scientific), lyophilized and stored at -20 °C for subsequent proteomic analysis.

Proteomics by NanoLC-MS/MS

Nano-LC-MS peptide analyses were performed on a Dionex UltiMate 3000 nano-HPLC system (Thermo Scientific, Bremen, Germany) coupled to a Thermo Scientific Q Exactive HF mass spectrometer. Samples were injected as 18 µL aqueous solutions containing 0.1% formic acid. Peptides were concentrated on a PepMap100 C18 trap column (300 µm i.d., 5 mm length, 5µm particles with 100 Å pore size) and separated on a fritless pulled fused-silica capillary column (100 µm i.d., 20 cm length) packed with 2.4 µm reversed-phase material (ReproSil-Pur C18-AQ with 120 Å pores). The gradient (solvent A: 0.1% formic acid; solvent B: 0.1% formic acid in 85% acetonitrile) was initially isocratic at 4% B for 4 min. Solvent B was then increased linearly to 35% within 53 min, and to 100% over 5 min, was kept at 100% for 5 min, and reduced linearly to 4% over 5 min. A flowrate of 300 nL/min was applied.

Molecular dynamic simulations of interactions of GMs with lipid membrane

Molecular dynamics simulations of graphene and GM nanosheets interacting with a model *E. coli* outer membrane were performed in an aqueous environment. Due to the complexity of the membrane structure, we used palmitoyloleoylphosphatidylethanolamine (POPE) lipids, which is one of the most abundantly found in Gram-negative bacteria and has been used as a model membrane for studying nanomaterials interaction with bacteria[8]. Interactions of the membrane with G1, G4 and G6 as well as pristine graphene was simulated. For G1, G4 and G6, the carboxyl groups have been randomly attached to the carbon atoms of the edge of the nanosheet while the other two functional groups are attached at random to carbon atoms on the basal plane. The adopted construction strategy complies with the model of Lerf-Klinowski which describes outcomes from oxidation processes[9].

The simulations have been carried out at atomistic resolution. A lipid membrane has been formed

by dissolving 340 POPE molecules in cubic box containing 31141 water molecules. The four nanosheets have the same dimensions of 4.92 nm × 1.99 nm. The Berger lipid force field has been adopted for the POPE molecules while water molecules have been represented by the SPC/E model.[10] The initial configuration of a single POPE molecule has been obtained from the ATB library.[11, 12] The force field for pure graphene is given in by Gong *et al.,*[13] while the OPLS-AA force field has been employed for the graphene oxide sheets.[14] All four nanosheets have been created using the GOPY tool.[15]

Equilibration of the dissolved lipids in water has been performed for 30 ns in the isothermal-isobaric (NPT) ensemble at 101.3 kPa and 300 K. The Nosé–Hoover thermostat and barostat was employed with coupling times of 0.1 and 1.0 ps.[16] A time step of 1 fs using the velocity-Verlet integration scheme has been used. A 1.1 nm atom-based cutoff for the summation of van der Waals interactions has been employed. The Coulomb interactions were computed using the Particle-Particle Particle-Mesh method.[17] Subsequently, the nanosheets were inserted either parallel or perpendicular to the surface of the lipid membrane at distances ranging from 1.5 nm to 0.1 nm with a step of 0.1 nm. Further equilibration for each system took place for 10 ns while keeping the distance between the nanosheets and the lipid membrane fixed. The simulations were performed with the LAMMPS code.[18]

The potential of mean force (PMF) between the lipid membrane and a nanosheet with a given orientation has been determined by a series of constrained simulations.[19] The constraining force, $F_C(r)$, along the direction normal to the surface of the membrane at distance r has been computed from the total forces exerted on the nanosheet, $\boldsymbol{F}_{sheet}$, and the membrane, $\boldsymbol{F}_{membrane}$:

$$F_C(r) = \frac{1}{2}\left\langle \frac{\boldsymbol{r}_1 - \boldsymbol{r}_2}{\|\boldsymbol{r}_1 - \boldsymbol{r}_2\|} \cdot \left(\boldsymbol{F}_{membrane}(\boldsymbol{r}_1) - \boldsymbol{F}_{sheet}(\boldsymbol{r}_2)\right) \right\rangle$$

where $\boldsymbol{r}_1$ and $\boldsymbol{r}_2$ are the center of mass position vectors of the membrane and the nanosheet and $\langle ... \rangle$ denotes time averaging. The PMF has been obtained by integrating the constraint force according to

$$W(r) = W(r_0) - \int_{r_0}^{r} F_C(r)dr$$

where $W(r_0)$ is the reference PMF value and it has been set to zero at distances larger than 1.6 nm between the lipid membrane and a nanosheet. In the production of the PMF profile, a series of constrained

simulations have been carried out with a decreasing step of 0.05 nm from 1.5 to 0.1 nm. Each simulation lasted for 4 ns. The first 3 ns constituted a pre-equilibrium stage, followed by a production run of 1 ns where the constrained force has been computed. The running average of the normalized force magnitude and potential energy exerted between the POPE membrane and the graphene nanosheet (size: 4.92 nm × 1.99 nm) at a distance of 0.3 nm between their axial center of mass coordinate were shown in the figure below. Both quantities are normalized by the average sampled between the 3.0 and 4.0 ns. It can be seen from these plots that both the force and the potential energy have reached their final value well before the 3 ns and that the system is well equilibrated.

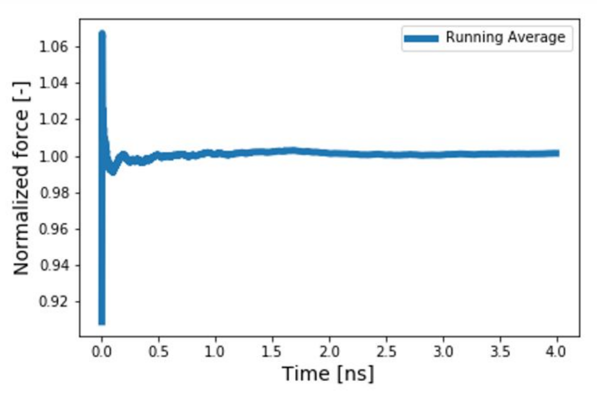

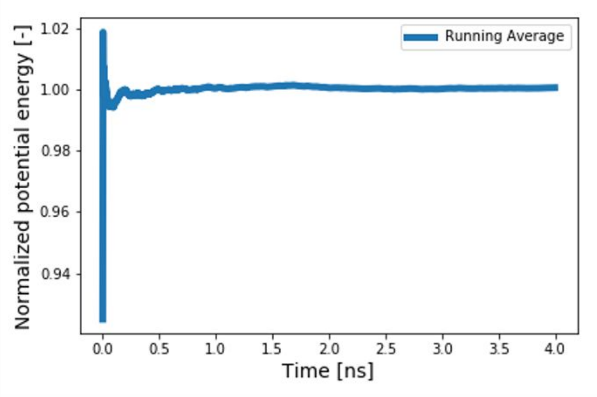

Rigidity of GMs by computational modeling

Insights into the rigidity of the graphene and graphene oxide nanosheets have been gained by carrying out tensile loading simulations on the four systems of interest (G1, G4, G6 and graphene) in vacuum, using the loading scheme of Vijayaraghavan and Zhang.[20] Initially, the systems have been equilibrated at 300 K using the same settings as before for 0.1 ns. Subsequently, a cycle consisting of (i) displacement of the boundary atoms along the longest dimension of the sheets with a constant outward velocity, (ii) equilibration of the system under tension while keeping the boundary atoms fixed and (iii) sampling of the mechanical response, has been performed 20 times. The displacement velocity used was 1.0 nm/ns. The duration of each of the three steps within a cycle was 0.1 ns. The stress tensor and the potential energy of the systems was recorded every 0.1 ps. The rigidity of the GMs was derived based on the variation of the potential energy per atom and force per atom of GMs. High value indicates high rigidity.

Figures and Tables

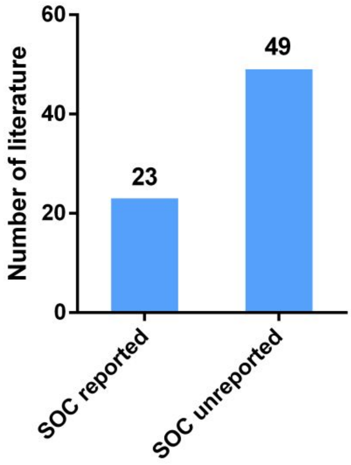

Fig. S1. A survey of the literature regarding the antibacterial effects of pristine GMs as well as GM composites that used pristine GMs as comparison since 2010. SOC indicates surface oxygen content measured by XPS. 72 paper was investigated [4, 8, 21-90]. Only 23 out of the 72 studies (32%) reported SOC. Subset of studies comparing the antibacterial effects of GO and rGO was shown in Table S1. Lack of SOC data renders the results from these studies difficult to compare.

Table S1. A survey of the studies which compared the antibacterial activity of pristine GO and rGO.

References	Bacterial species	Exposure method	Antibacterial activity	SOC reported	Lateral size (μm)	Thickness (nm)	Synthesis method
Liu *et al.*[24]	*Escherichia coli*	Suspension exposure in saline and cell colony counting	GO > rGO	No	GO: 0.31<br>rGO: 2.75	1	GO: Hummers; rGO: hydrazine reduction of GO
Kurantowicz *et al.*[85]	*Listeria monocytogenes, Salmonella enterica*	Suspension exposure in saline and cell colony counting	GO > rGO	No	GO: 1.27<br>rGO: 2.53	rGO: few layer<br>GO: single layer;<br>Value not given	GO: Hummers;<br>rGO: reduction of GO by ammonium iodide, sodium hypophosphite and sodium sulfite mixture
Gurunathan *et al.*[86]	*Pseudomonas aeruginosa.*	Suspension exposure in saline	GO > rGO	No	Only hydrodynamic sizes were given:<br>GO: 0.53<br>rGO: 3.4	Not given	GO: Hummers;<br>rGO: reduction of GO by betamercaptoethanol

Gurunathan *et al.*[38]	*Escherichia coli*	Suspension exposure in saline and cell colony counting	GO > rGO	No	Only hydrodynamic sizes were given: GO: 0.48 rGO: 2.9	Not given	GO: Hummers; rGO: reduction of GO by dithiothreitol
Chen *et al.*[87]	*Xanthomonas oryzae pv*	Suspension exposure in saline and cell colony counting	GO > rGO	No	Only hydrodynamic sizes were given: 0.3~0.6; GO < rGO	Not given	GO: Hummers; rGO: hydrazine reduction of GO
Hu *et al.*[4]	*Escherichia coli*	Suspension exposure in saline for nanosheet; airbone bacterial test for GO/rGO paper	GO > rGO	No	AFM image provided but sizes were not given	GO: 1.1 rGO: 1	GO: Hummers; rGO: hydrazine reduction of GO
Akhavan *et al.*[82]	*Escherichia coli*	Drop test on GO/RGO film on Si substrate and cell colony counting	GO < rGO	Yes [a]	AFM image provided but sizes were not given	~ 1	GO: Hummers; rGO: reduction of GO by *Escherichia coli*
Akhavan *et al.*[21]	*Escherichia coli* & *S. aureus*	Drop test on GO/RGO nanowalls	GO < rGO	Yes [b]	Not given	Not given	GO: Hummers; rGO: hydrazine reduction of GO nanowalls
Dutta *et al.*[50]	*Enterobacter spp.*	Suspension assay in nutrient broth	GO < rGO	No	Not given	Not given	GO: Hummers; rGO: reduction of GO by hydrazine hydrate, sodium borohydride, hypophosphorous acid or sodium dithionite
Barrios *et al.*[88]	*Escherichia coli*	Suspension exposure in saline and cell colony counting	GO < rGO	Yes [c]	GO: 1.19 rGO: 1.11	Not given	GO: Hummers; rGO: thermal anneal of GO
		**Note:** *SOC dependent test performed; higher SOC leads to lower antibacterial effects*					
Zhao *et al.*[89]	*Escherichia coli* & *S. aureus*	Suspension exposure in LB medium and cell colony counting	GO < rGO	Yes [d]	Not given	GO: 1.22-1.7 rGO: 0.94-1.21	GO: Hummers; rGO: UV irradiation of GO suspension
		**Note:** *SOC dependent test performed; contradictory results obtained: higher SOC leads to higher antibacterial effects;*					
Hou *et al.*[90]	*Escherichia coli & Bacillus subtilis*	Suspension exposure in MMD medium and colony counting	GO < rGO	Yes [e]	GO: 0.055μm^2; rGO: 0.041 μm^2	GO: 0.9; rGO: 2.2	GO: Hummers; rGO: solar irradiation of GO suspension
			GO > rGO		GO: 0.055μm^2; rGO: 0.005 μm^2		GO: Hummers; rGO: solar irradiation of GO suspension with presence of H_2O_2

Note: [a] SOC was not provided; however, C~C/C~O (sp^2 carbon and C~O bond) ratio was given. The values were 0.46 and 1.05 for GO and rGO, respectively. [b] SOC was not provided; however, C~C/C~O (sp^2 carbon and C~O bond) ratio was given. The values were 0.4 and 2.5 for GO and rGO, respectively. [c] SOC was provided (0.12 ~ 0.33). [d] The SOC was not given; however, it can be calculated based on the provided O/C ratio. The calculated SOC was 0.24~0.37 for GO, and 0.21~0.25 for rGO, respectively. [e] The SOC was not given; however, it can be calculated based on the provided O/C ratio. The calculated SOC was 0.35 for the GO, and 0.35~0.32 and 0.34~0.28 for the rGO obtained by solar irradiation without

and with the presence of H_2O_2, respectively.

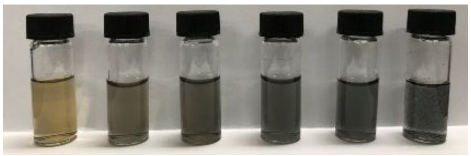

Fig. S2. Digital photographs of GM suspensions (100 mg/L). From left to right: G1, G2, G3, G4, G5 and G6.

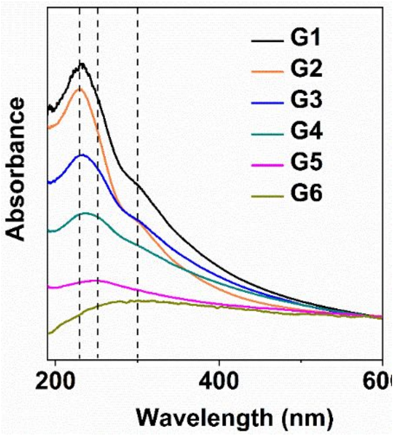

Fig. S3. UV-vis spectra of GM suspensions at 100 mg/L. The absorbance peak of the GMs shifts from 230 nm to 250 nm, and the slight peak at 300 nm disappears with decreasing SOC.

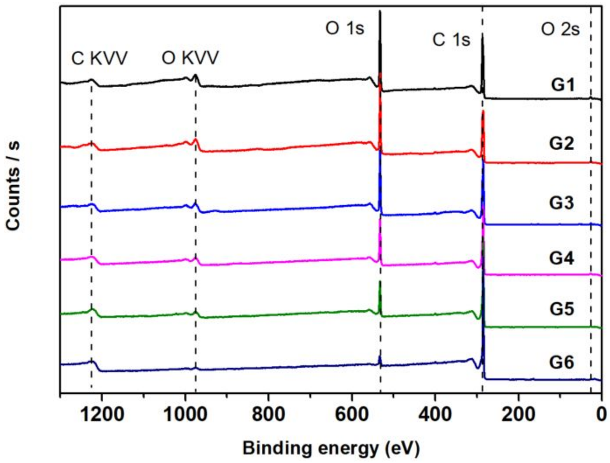

Fig. S4. XPS survey spectra of the GMs. The data show C and O as the main constituents with no

impurities.

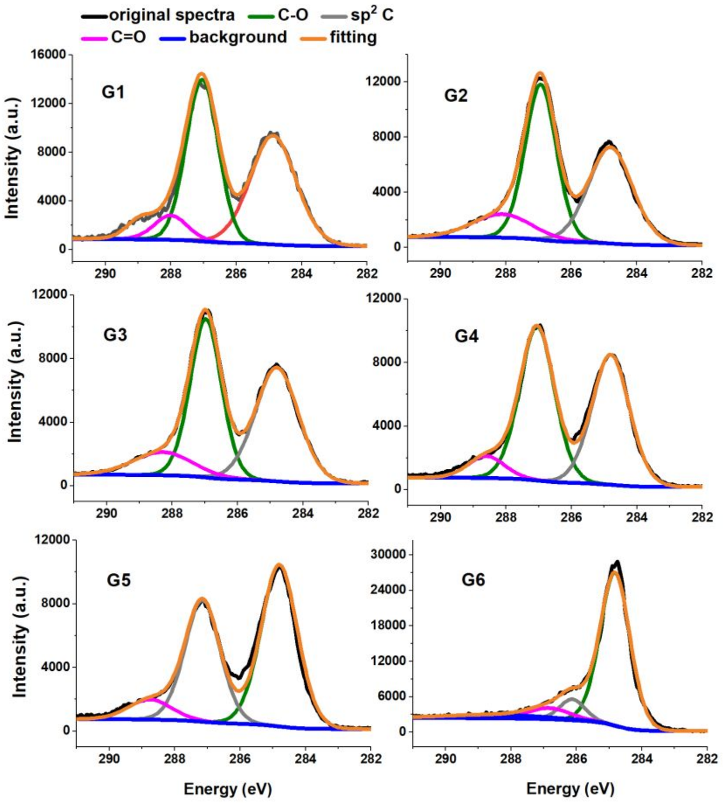

Fig. S5. High resolution C1s XPS spectra of GMs. C1s peaks of XPS spectra were deconvoluted to get the fraction of different carbon functional groups. The intensity of the C-C/C=C peak relative to C-O/C=O gradually increased as the decrease of SOC. Typical peaks centered at binding energy of 284.8 eV, 286.5 eV, 287.9 eV, and 289.0 eV were respectively ascribed to C-C/C=C bonds, C-O (epoxy and alkoxy), C=O, and O=C-OH functional groups.

Table S2. Fraction of different chemical species of C in GMs with different oxygenated groups and the SOC obtained by fitting of the C1s XPS spectra. SOC (O %/100) indicates the fraction of O in total atoms. The superscript ^{S} indicates the atomic percent of C and O in the GMs after 2 years of storage in a vacuum desiccator. Data shows that the O% did not change after long term storage.

	C %	O %	% sp^2 C	%C-O	%C=O	% COOH	SOC	C % ^{S}	O % ^{S}
G1	65.4	34.6	39.3	49.53	8.03	3.14	0.346	65.0	35.0
G2	66.4	33.6	40.79	48.14	6.81	4.26	0.336	66.6	33.8
G3	68.7	31.3	44.12	43.56	9.58	2.74	0.313	68.3	31.7

G4	70.4	29.6	48.57	40.69	3.63	7.12	0.296	70.5	29.5
G5	73.7	26.3	53.28	32.39	7.81	6.52	0.263	73.2	26.8
G6	92.2	7.8	70.79	15.79	4.43	8.99	0.078	92.5	7.5

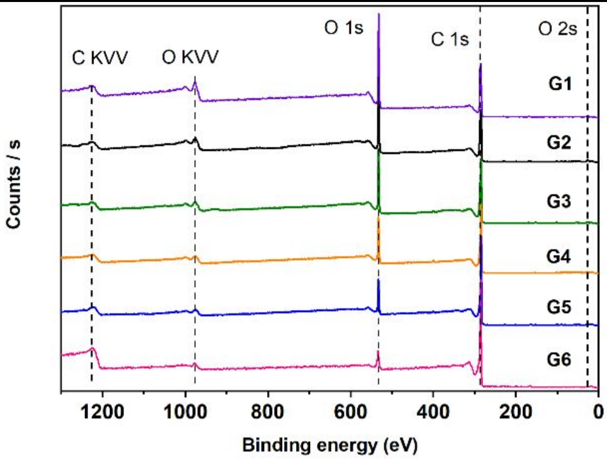

Fig. S6. XPS survey spectra of GMs after 2 years of storage in vacuum desiccator. The obtained atomic percentage of C and O did not change after storage (Table S2).

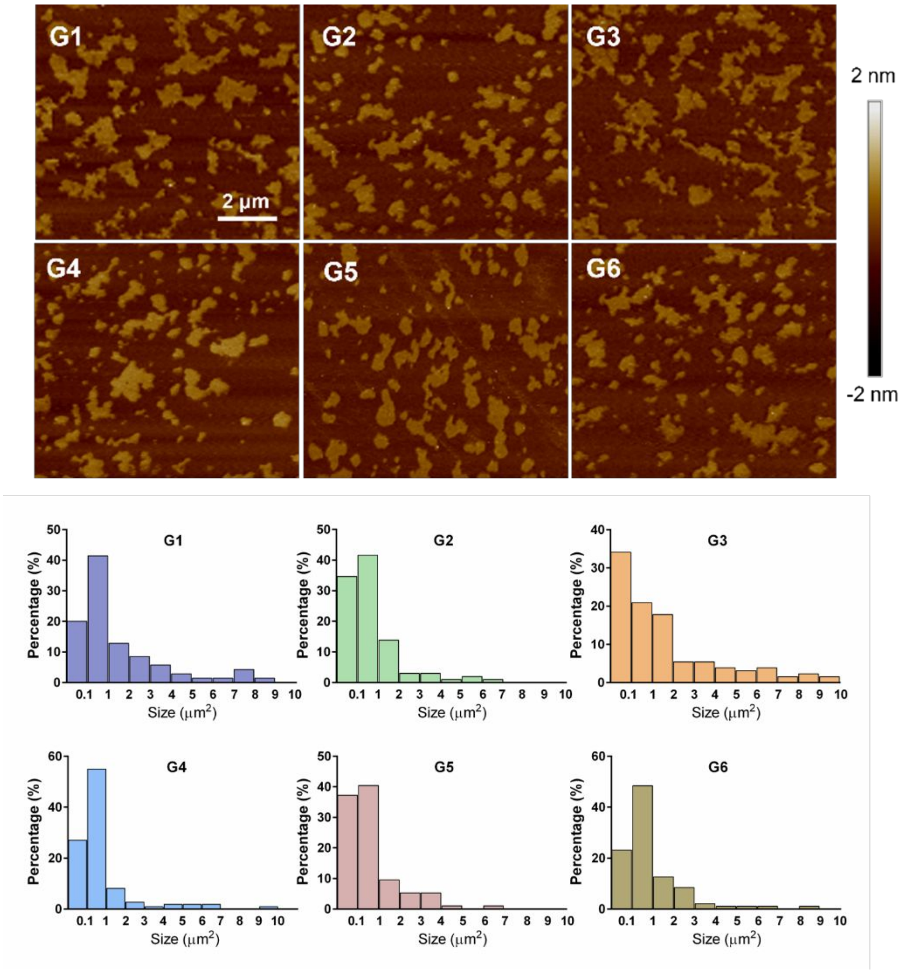

Fig. S7. AFM images and size distribution of the pristine GMs. The white scale bar indicates 2 µm and is applicable to all six images. The sheet area (µm²) of the GMs is shown as a histogram by counting more

than 100 sheets for each GM. The sheet area distributions of the GMs are all similar. The thicknesses were ~ 1 nm for all the GMs.

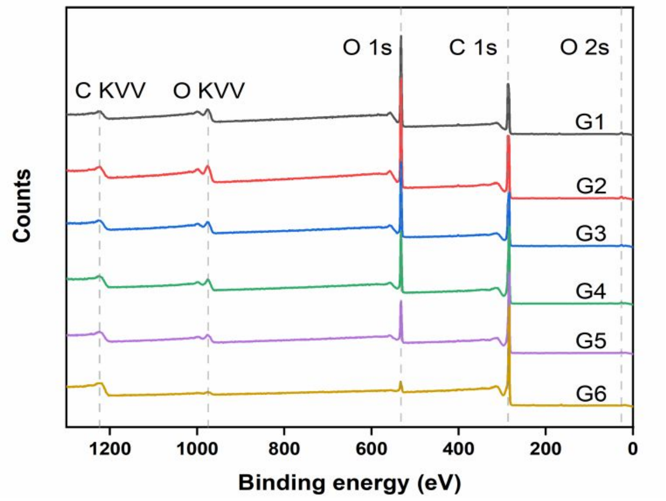

Fig. S8 XPS survey spectra of the GMs. The data show C and O as the main constituents with no impurities.

Table S3. Fraction of different chemical species of C in GMs with different oxygenated groups and the SOC. SOC (O%/100) indicates the fraction of O in total atoms. Data shows that the O% did not change between different batches, suggesting that the SOC could be well controlled.

	C %	O %	% sp^2 C	% C-O	% C=O	% COOH	SOC
G1	64.3	35.7	39.1	48.24	9.17	3.49	0.357
G2	67.5	32.5	41.85	47.31	7.15	3.69	0.325
G3	69.3	30.7	44.34	43.79	9.47	2.4	0.307
G4	71.2	28.8	47.79	41.53	3.88	6.8	0.288
G5	74.3	25.7	54.15	33.48	7.27	5.1	0.257
G6	93.1	6.9	71.22	14.84	5.18	8.76	0.069

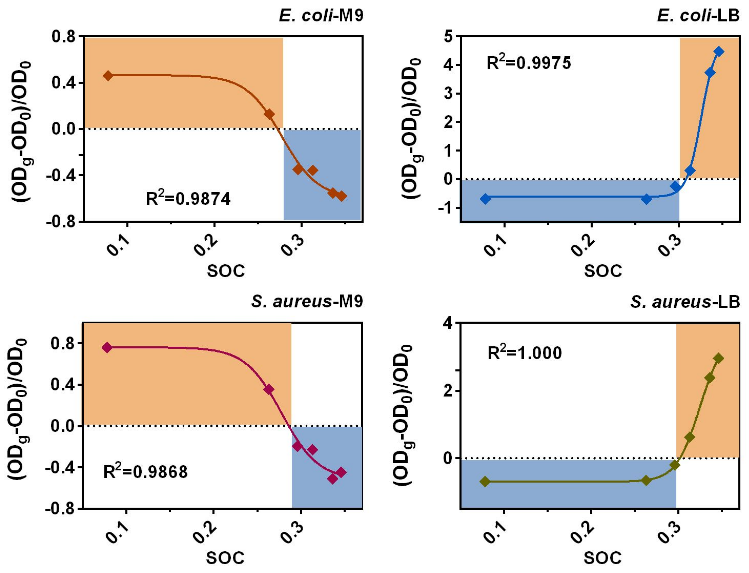

Fig. S9. Logistic regression analysis of the fraction of surface oxygen on the GMs (SOC, x-axis) with the percent of loss of viability (OD_{540}) in M9 or LB medium (y-axis). OD_0 indicates OD_{540} values in control group. OD_g indicates OD_{540} values after exposed to GO suspension for 48 h.

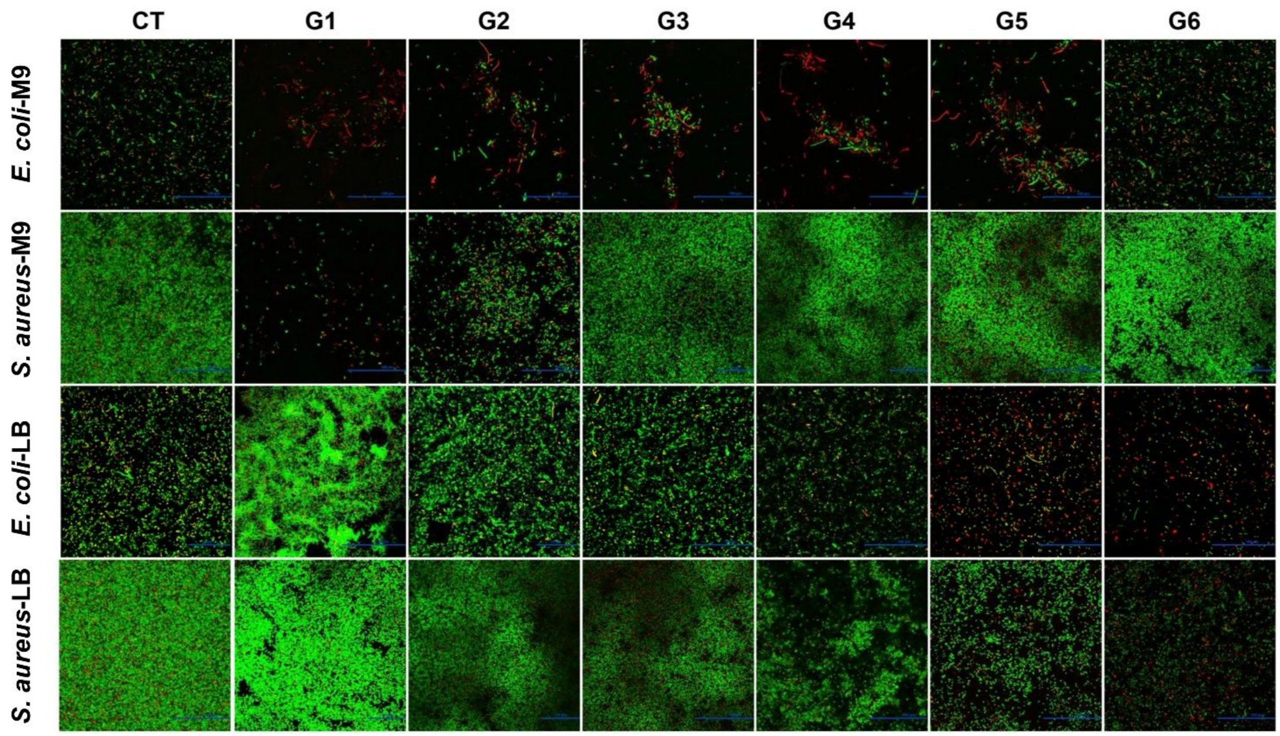

Fig. S10. CLSM micrographs of biofilm on glass slides after incubation with and without GMs in M9 or LB medium for 48 h. Live cells were stained in green and dead cells were stained in red. Images were obtained by overlaying the red and green channels. The blue scale bar indicates 100 μm.

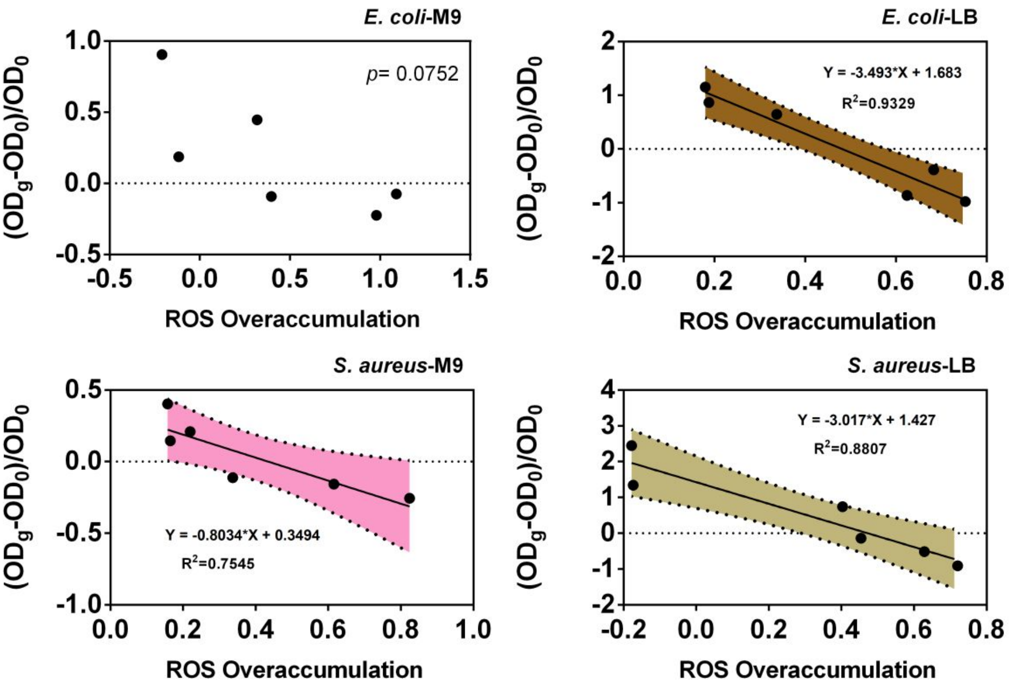

Fig. S11. Intracellular reactive oxygen species (ROS) content was correlated with the percent of loss of cell viability (OD_{600}) in the two media.

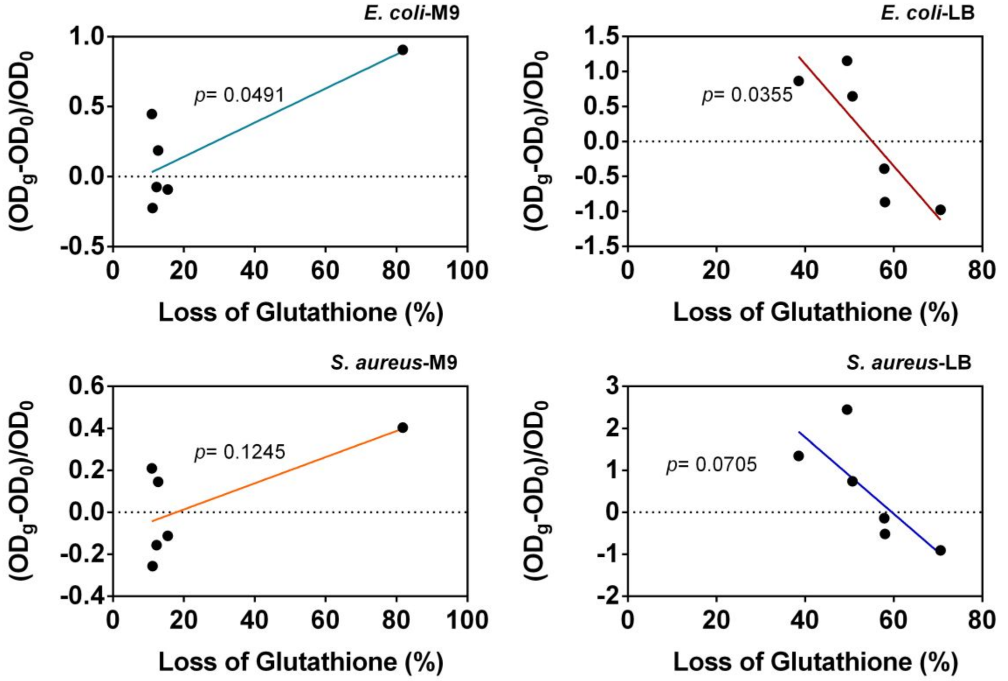

Fig. S12. The GSH oxidative potential of GMs was not correlated with the percent of loss of cell viability (OD_{600}).

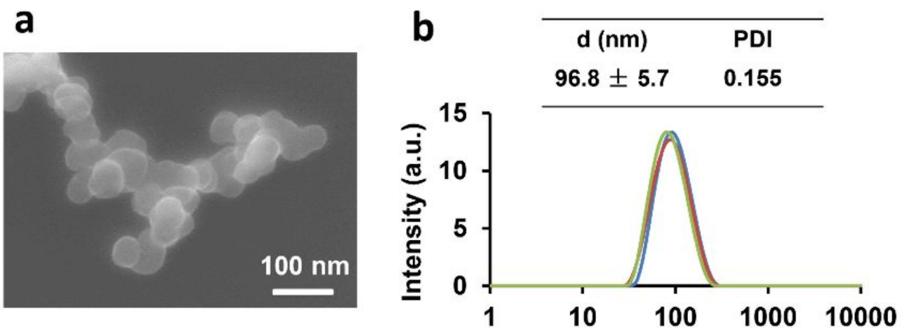

Fig. S13. SEM image (**a**) and hydrodynamic size (**b**) of the lipid vesicles. The average sizes calculated from the SEM image is 51 nm. The hydrodynamic size is 96.8 nm with a polydispersity index of 0.155, indicating the vesicle suspension (100 mg/mL) is monodispersed.

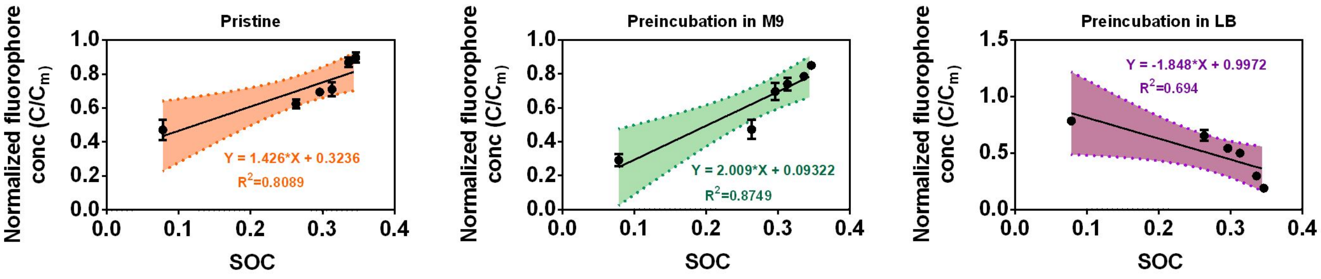

Fig. S14. The leaking of fluorophore was correlated with SOC after 3 h incubation with GMs, indicative of membrane damage increasing with SOC in the absence of a protein corona but decreasing with increasing SOC in the presence of the corona.

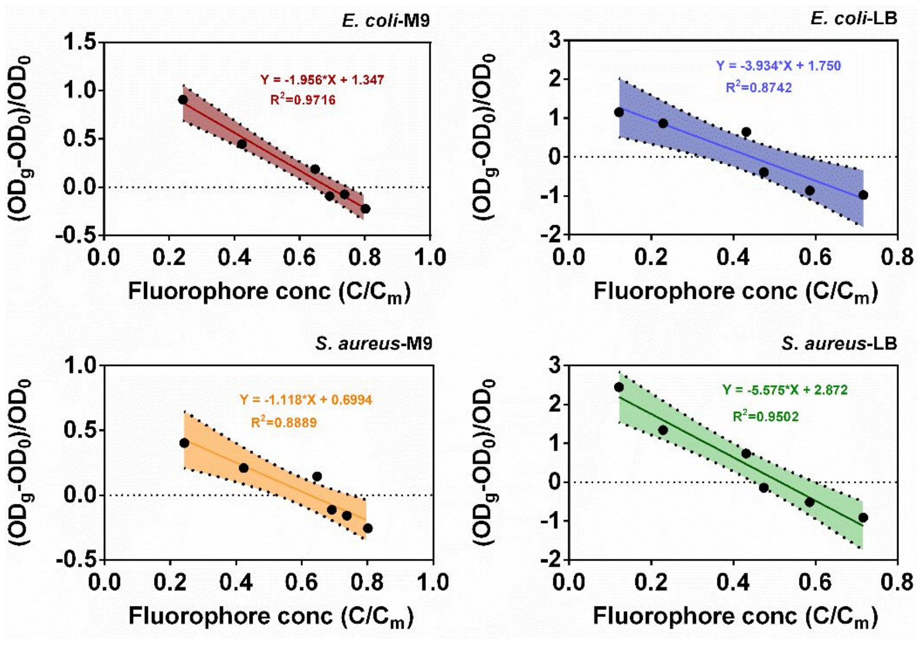

Fig. S15. The fraction of the leaked fluorophore at 3 h was correlated with the percent of loss of viability

(OD_{600}) at 48 h exposure to GMs, suggesting that physical disruption is the main contributor to the differential antibacterial activity of GMs with different SOC

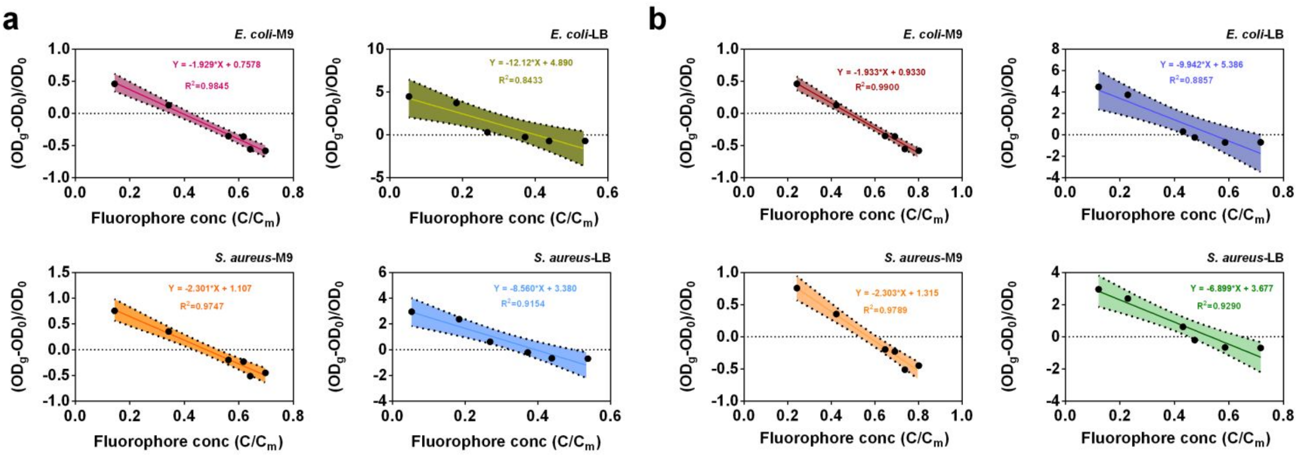

Fig. S16. The ability of physical disruption of was correlated with the percent of loss of cell viability (biofilm biomass, OD_{540}) after 48 h of exposure to GMs. **a**, leaking of fluorophore after 1 h; **b**, leaking of fluorophore after 3 h.

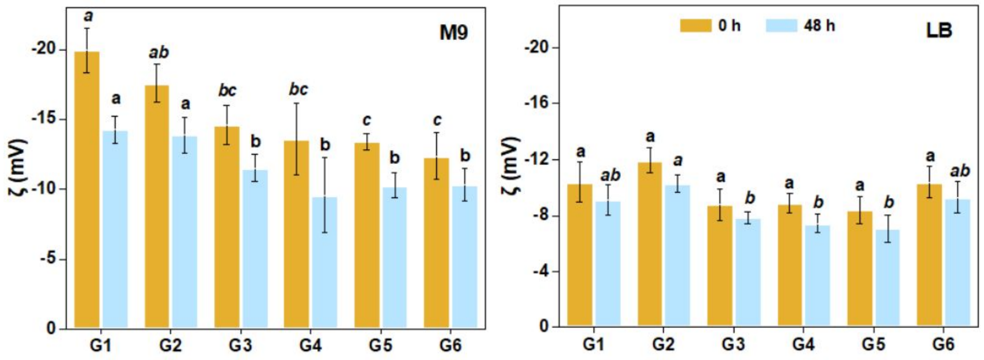

Fig. S17. Zeta (ζ) potential of GMs in M9 and LB medium (100 mg/L) at 0 and 48 h after preparation. The ζ potential was higher for GMs with higher SOC in M9; the difference was diminished after 48 h. The ζ potentials were similar for all GMs in LB.

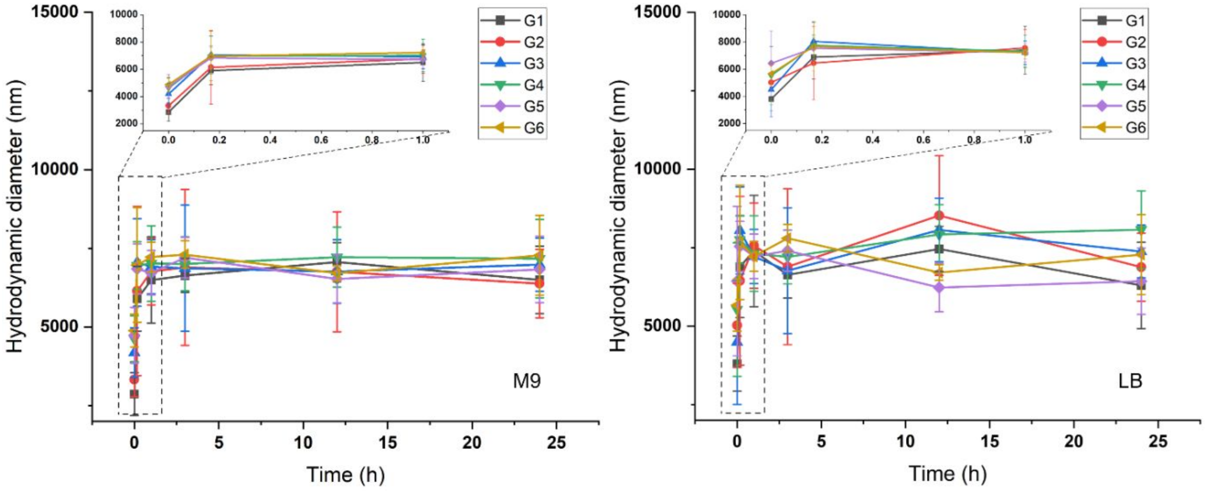

Fig. S18. Hydrodynamic diameters of GMs in M9 (**a**) and LB (**b**) medium (100 mg/L) over the course of 24 h. The diameters were recorded at 0, 10 min, 1 h, 3 h, 12 h and 24 h after dispersion preparation. The zoomed insert plots show the magnified plot from time 0 to 1h.

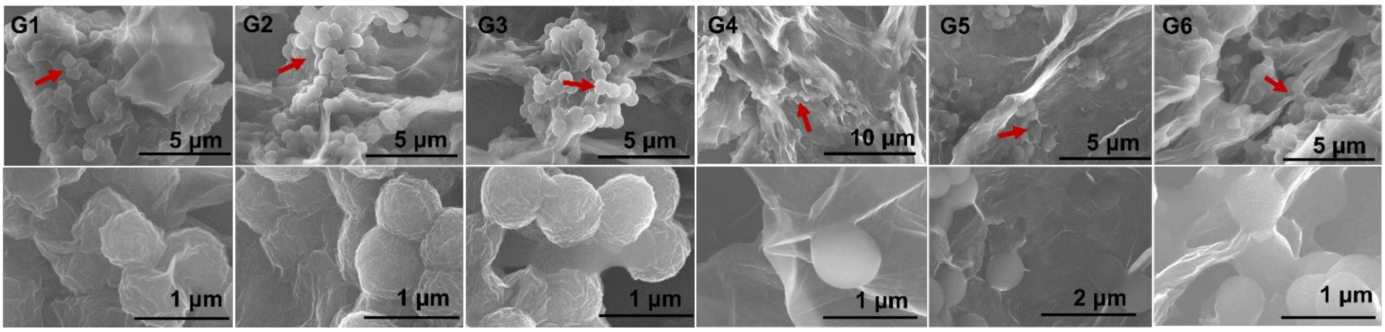

Fig. S19 SEM images of *S. aureus* after treatment with GMs in LB media. Arrow in the upper panel indicates the region magnified in the lower panel.

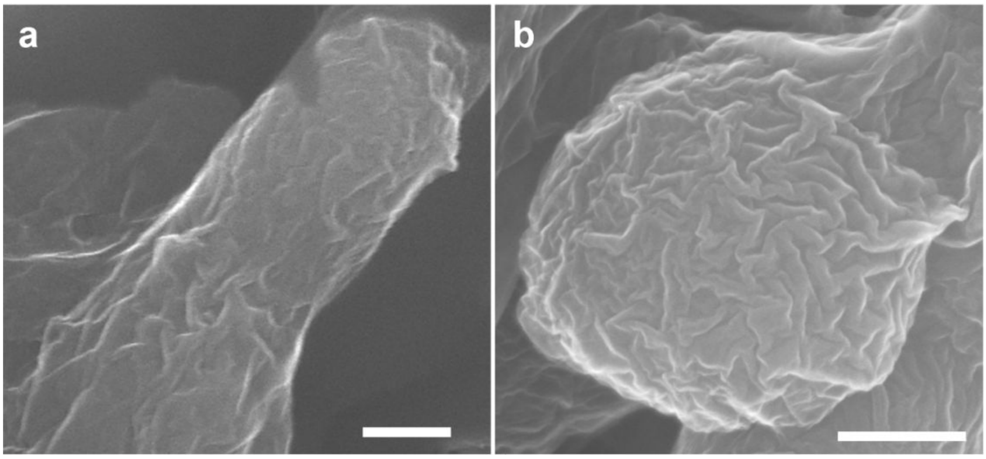

Fig. S20. Magnified images of G1 treated *E. coli* (**a**) and *S. aureus* (**b**) in M9 media showing the wrinkled surface morphology. Scale bar indicates 200 nm.

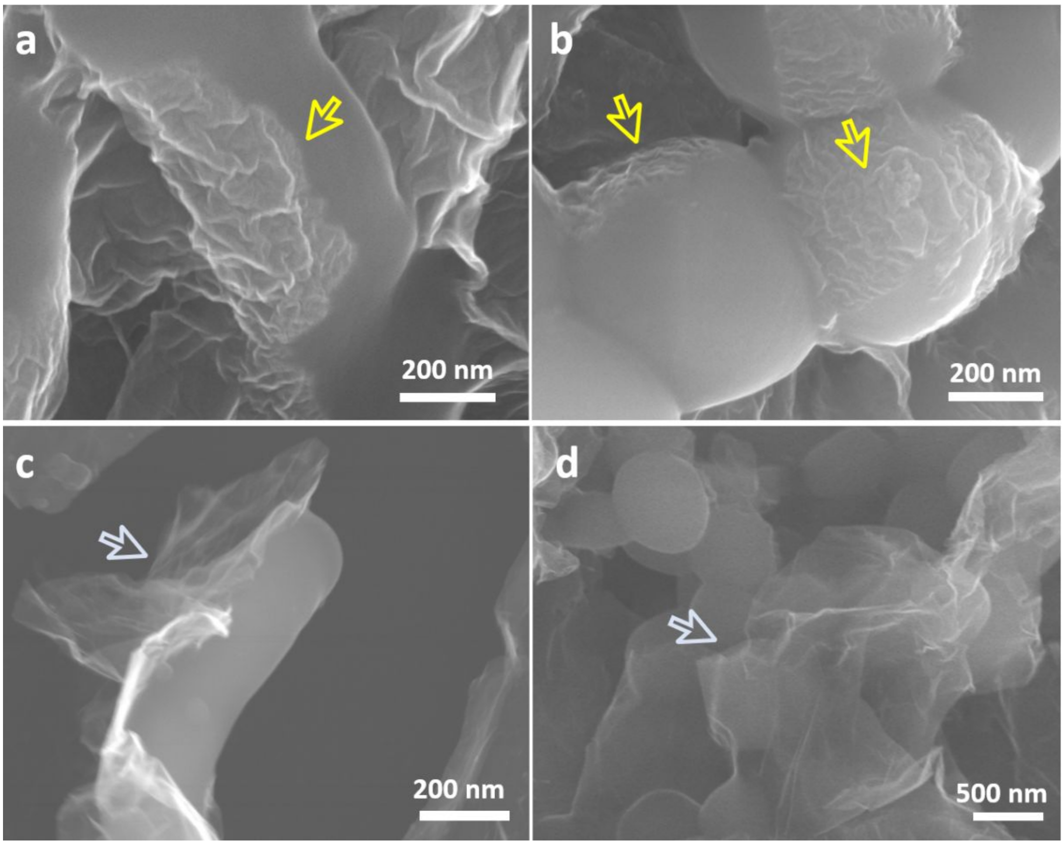

Fig. S21. a, b, SEM image of small sized G1 fragment attaching closely in parallel to bacterial surface. Wrinkled morphology can be observed even the surface was only partially covered. **c, d,** SEM images of G6 loosely covering the bacterial surface with no wrinkled surface morphology.

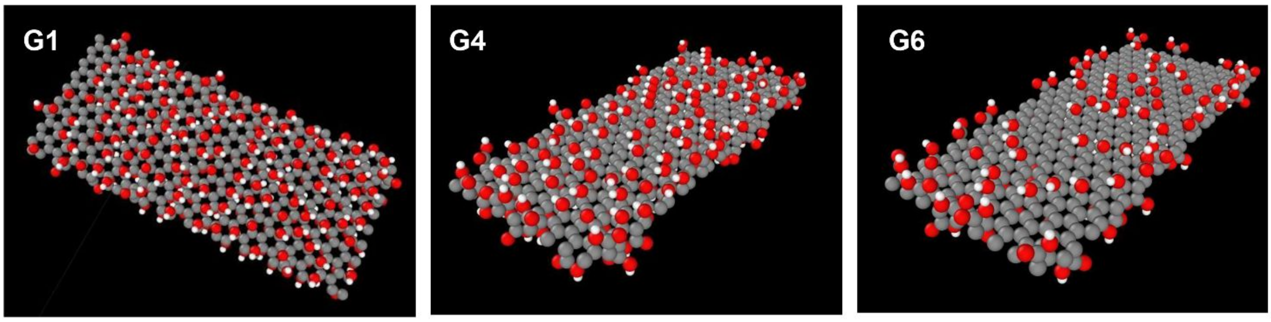

Fig. S22. G1, G4 and G6 graphene sheets used for MD simulation. Gray, red and white indicate carbon, oxygen and hydrogen atoms.

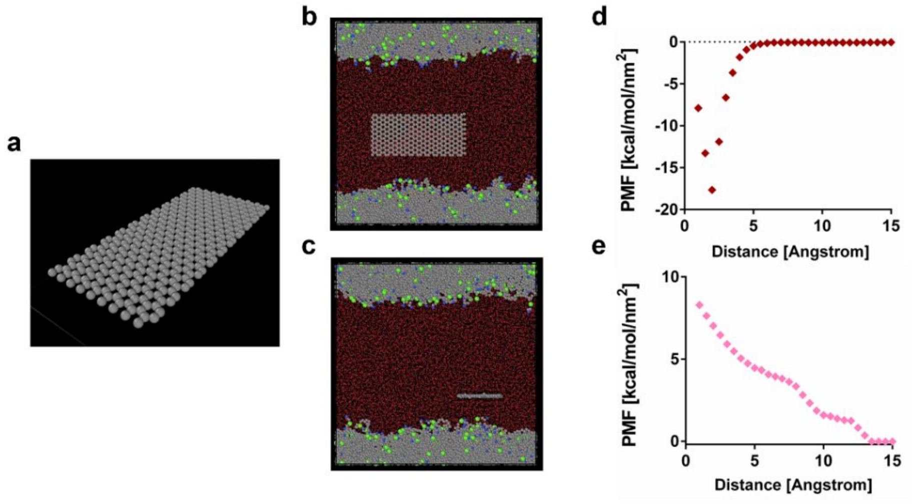

Fig. S23. **a**, Graphene sheets used for MD simulation. **b**, **c**, Representative configurations of graphene nanosheet aligned perpendicular (**b**) or parallel (**c**) to the surface of the lipid membrane during the constrained MD simulations. For clarity, the hydrogen atoms are hidden while the oxygen atoms of all water molecules have a reduced size. the hydrogen atoms are hidden while the oxygen atoms of all water molecules have a reduced size. We have used a gray / blue / green color for the carbon / nitrogen / phosphorus atoms in the system. **d**, **e**, Potential of mean force as a function of distance for a perpendicularly (in **d**) or parallelly (in **e**) oriented graphene nanosheet to the surface of the lipid membrane. The negative PMF at a perpendicular orientation suggests there is attractive force between graphene and the lipid membrane. However, strong repelling force (positive PMF) was observed when the orientation is parallel.

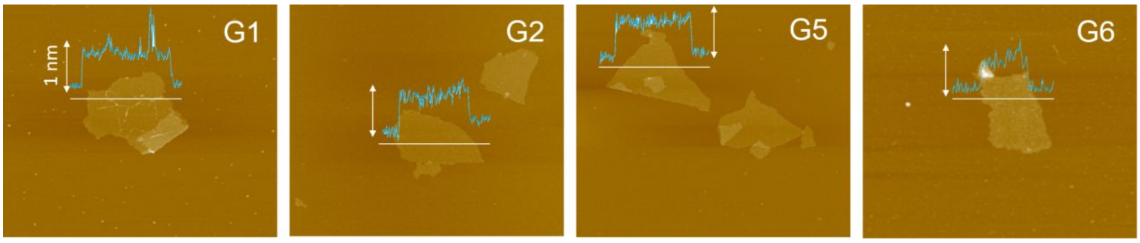

Fig. S24. AFM images of GMs after incubation in M9 medium for 3 h. Arrows indicate 1 nm for all figures. The heights of the GMs were ~ 1 nm which is similar with the pristine GMs.

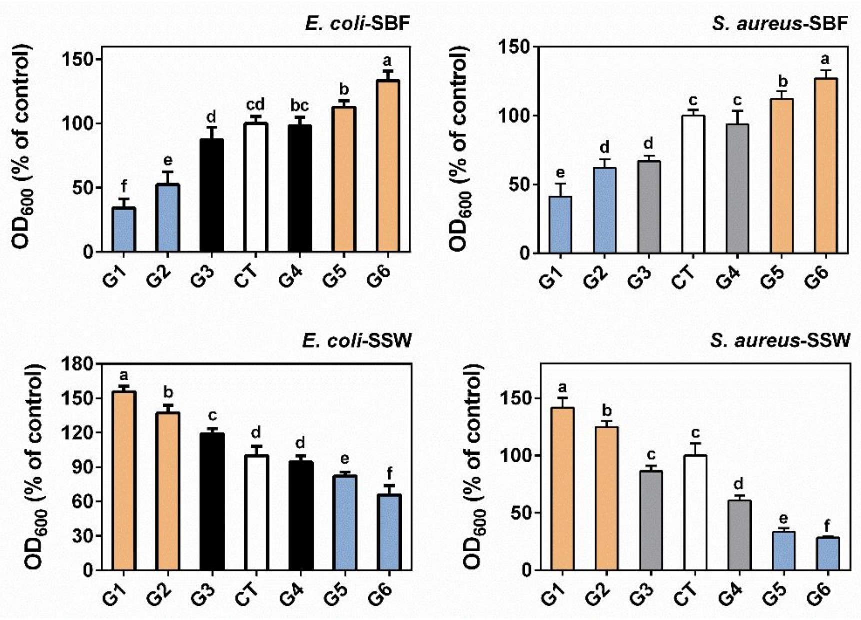

Fig. S25. Antibacterial activity of GMs with different SOC against *E. coli* or *S. aureus* in SBF and SWW. The viability of the bacterial cells was expressed as % of control. Different lowercase letters indicate significant difference at $p < 0.05$ (n = 6, mean ± s.d.).

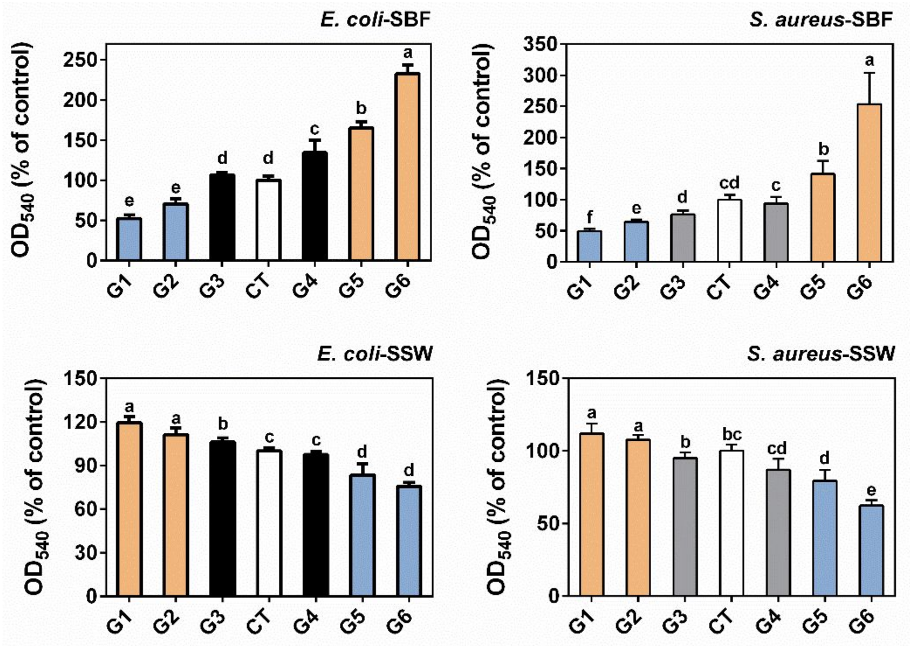

Fig. S26. Bacterial biofilm formation measured by a crystal violet staining method after interaction with GMs in SBF and SWW for 48 h. Different lowercase letters indicate significant difference at $p < 0.05$ (n = 6, mean ± s.d.).

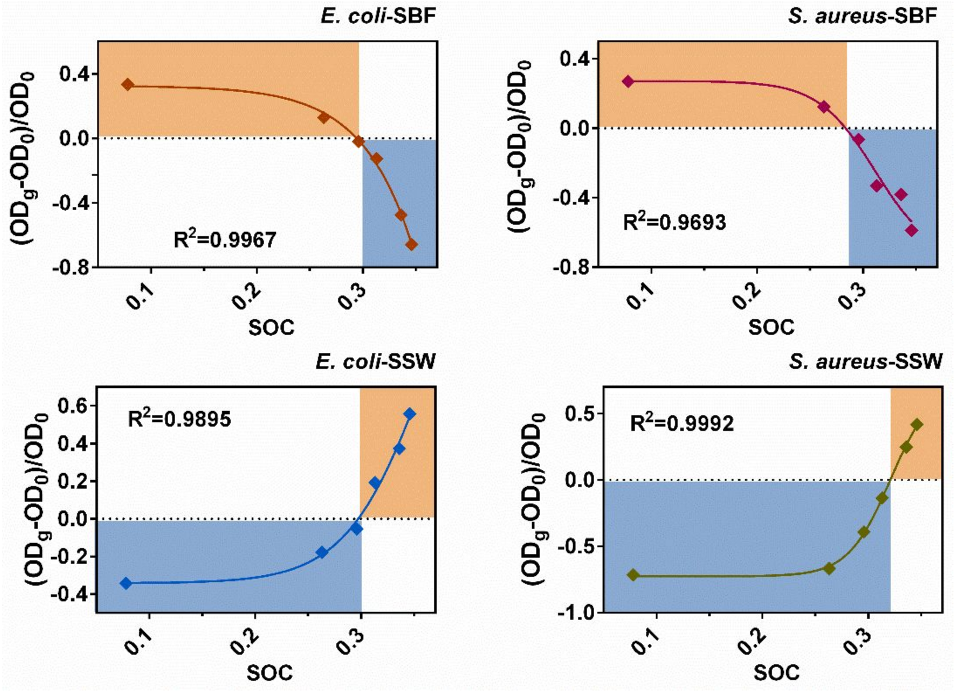

Fig. S27. Logistic regression analysis of the fraction of surface oxygen on the GMs (SOC, x-axis) with the percent loss of cell viability (data from Fig. S23) in SBF or SSW medium (y-axis). OD_0 indicates OD_{600} values in control group. OD_g indicates OD_{600} values after exposed to GMs suspension for 48 h.

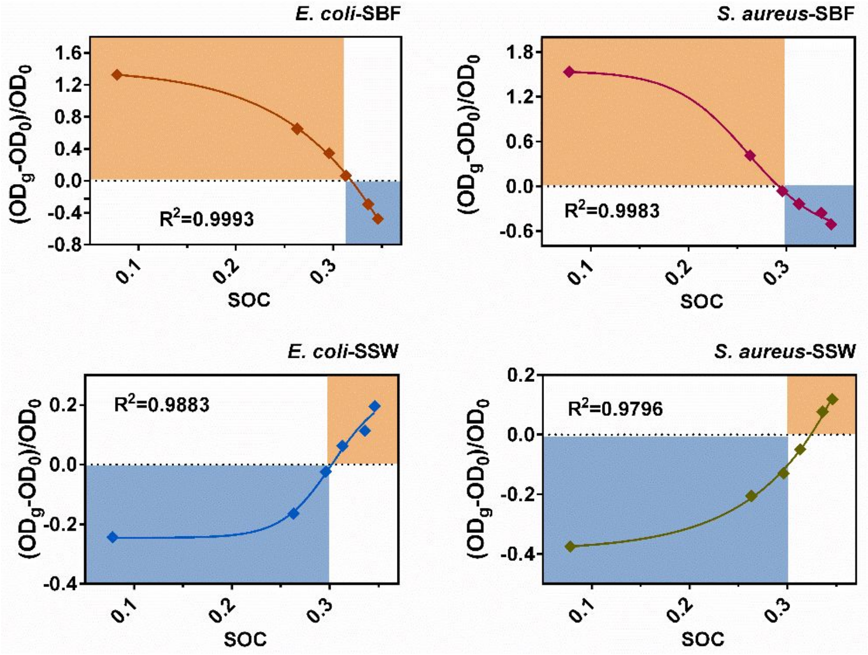

Fig. S28. Logistic regression analysis of the fraction of surface oxygen on the GMs (SOC, x-axis) with the percent of loss of biofilm density (data from Fig. S24) in SBF or SSW medium (y-axis). OD_0 indicates OD_{540} values in control group. OD_g indicates OD_{540} values after exposed to GMs suspension for 48 h.

Table S4. Complete list of the absorbed proteins on GMs obtained by proteomics (**Table S4** attached as a separate file).

References

1. Hummers WS, Offeman RE. Preparation of Graphitic Oxide. *Journal of the American Chemical Society* 1958, **80**(6)**:** 1339-1339.
2. Majeed W, Bourdo S, Petibone DM, Saini V, Vang KB, Nima ZA*, et al.* The Role of Surface Chemistry in the Cytotoxicity Profile of Graphene. *Journal of applied toxicology : JAT* 2017, **37**(4)**:** 462-470.
3. Fernández-Merino MJ, Guardia L, Paredes JI, Villar-Rodil S, Solís-Fernández P, Martínez-Alonso A*, et al.* Vitamin C Is an Ideal Substitute for Hydrazine in the Reduction of Graphene Oxide Suspensions. *The Journal of Physical Chemistry C* 2010, **114**(14)**:** 6426-6432.
4. Hu W, Peng C, Luo W, Lv M, Li X, Li D*, et al.* Graphene-Based Antibacterial Paper. *ACS Nano* 2010, **4**(7)**:** 4317-4323.
5. Kokubo, T, and Hiroaki T. How Useful is SBF in Predicting *in vivo* Bone Bioactivity?. *Biomaterials* 2006, **27**(15): 2907-2915.
6. Taştan BE and Gönül D. Biodegradation of Pesticide Triclosan by A. Versicolor in Simulated Wastewater and Semi-synthetic Media. *Pesticide Biochemistry and Physiology* 2015, **118**: 33-37.
7. Guo Z, Xie C, Zhang P, Zhang J, Wang G, He X*, et al.* Toxicity and Transformation of Graphene Oxide and Reduced Graphene Oxide in Bacteria Biofilm. *Science of the Total Environment* 2017, **580:** 1300-1308.
8. Tu Y, Lv M, Xiu P, Huynh T, Zhang M, Castelli M*, et al.* Destructive Extraction of Phospholipids from Escherichia Coli Membranes by Graphene Nanosheets. *Nature Nanotechnology* 2013, **8**(8)**:** 594-601.
9. Lerf A, He H, Forster M, Klinowski J. Structure of Graphite Oxide Revisited. *The Journal of Physical Chemistry B* 1998, **102**(23)**:** 4477-4482.
10. Berger O, Edholm O, Jähnig F. Molecular Dynamics Simulations of a Fluid Bilayer of

Dipalmitoylphosphatidylcholine at Full Hydration, Constant Pressure, and Constant Temperature. *Biophysical journal* 1997, **72**(5)**:** 2002-2013.

11. Malde AK, Zuo L, Breeze M, Stroet M, Poger D, Nair PC*, et al.* An Automated Force Field Topology Builder (ATB) and Repository: Version 1.0. *Journal of Chemical Theory and Computation* 2011, **7**(12)**:** 4026-4037.

12. Koziara KB, Stroet M, Malde AK, Mark AE. Testing and Validation of the Automated Topology Builder (ATB) Version 2.0: Prediction of Hydration Free Enthalpies. *Journal of computer-aided molecular design* 2014, **28**(3)**:** 221-233.

13. Gong X, Li J, Lu H, Wan R, Li J, Hu J*, et al.* A Charge-Driven Molecular Water Pump. *Nature Nanotechnology* 2007, **2**(11)**:** 709-712.

14. Tang H, Liu D, Zhao Y, Yang X, Lu J, Cui F. Molecular Dynamics Study of the Aggregation Process of Graphene Oxide in Water. *The Journal of Physical Chemistry C* 2015, **119**(47)**:** 26712-26718.

15. Muraru S, Burns JS, Ionita M. GOPY: A Tool for Building 2D Graphene-Based Computational Models. *SoftwareX* 2020, **12:** 100586.

16. Frenkel D, Smit B. *Understanding molecular simulation: from algorithms to applications*, vol. 1. Elsevier, 2001.

17. Hockney RW, Eastwood JW. *Computer simulation using particles*. crc Press, 2021.

18. Plimpton SJJocp. Fast Parallel Algorithms for Short-Range Molecular Dynamics. 1995, **117**(1)**:** 1-19.

19. Carter E, Ciccotti G, Hynes JT, Kapral RJCPL. Constrained Reaction Coordinate Dynamics for the Simulation of Rare Events. 1989, **156**(5)**:** 472-477.

20. Venkatesh V and Zhang L. Effective Mechanical Properties and Thickness Determination of Boron Nitride Nanosheets Using Molecular Dynamics Simulation. *Nanomaterials* 2018, **8**(7): 546.

21. Akhavan O, Ghaderi EJAn. Toxicity of Graphene and Graphene Oxide Nanowalls Against Bacteria. 2010, **4**(10)**:** 5731-5736.

22. Santos CM, Tria MCR, Vergara RAMV, Ahmed F, Advincula RC, Rodrigues DF. Antimicrobial Graphene Polymer (PVK-GO) Nanocomposite Films. *Chemical Communications* 2011, **47**(31)**:** 8892-8894.

23. Das MR, Sarma RK, Saikia R, Kale VS, Shelke MV, Sengupta P. Synthesis of Silver Nanoparticles in an Aqueous Suspension of Graphene Oxide Sheets and Its Antimicrobial Activity. *Colloids and Surfaces B: Biointerfaces* 2011, **83**(1)**:** 16-22.

24. Liu S, Zeng TH, Hofmann M, Burcombe E, Wei J, Jiang R*, et al.* Antibacterial Activity of Graphite, Graphite Oxide, Graphene Oxide, and Reduced Graphene Oxide: Membrane and Oxidative Stress. *ACS Nano* 2011, **5**(9)**:** 6971-6980.

25. Ruiz ON, Fernando KAS, Wang B, Brown NA, Luo PG, McNamara ND*, et al.* Graphene Oxide: A Nonspecific Enhancer of Cellular Growth. *ACS Nano* 2011, **5**(10)**:** 8100-8107.

26. Bao Q, Zhang D, Qi P. Synthesis and Characterization of Silver Nanoparticle and Graphene Oxide Nanosheet Composites as a Bactericidal Agent for Water Disinfection. *Journal of Colloid and Interface Science* 2011, **360**(2)**:** 463-470.

27. Sreeprasad TS, Maliyekkal MS, Deepti K, Chaudhari K, Xavier PL, Pradeep T. Transparent, Luminescent, Antibacterial and Patternable Film Forming Composites of Graphene Oxide/Reduced Graphene Oxide. *ACS Applied Materials & Interfaces* 2011, **3**(7)**:** 2643-2654.

28. Mondal T, Bhowmick AK, Krishnamoorti R. Chlorophenyl Pendant Decorated Graphene Sheet As a Potential Antimicrobial Agent: Synthesis and Characterization. *Journal of Materials Chemistry* 2012, **22**(42)**:** 22481-22487.

29. Mejías Carpio IE, Santos CM, Wei X, Rodrigues DF. Toxicity of a Polymer–graphene Oxide Composite Against Bacterial Planktonic Cells, Biofilms, and Mammalian Cells. *Nanoscale* 2012, **4**(15)**:** 4746-4756.

30. Krishnamoorthy K, Veerapandian M, Zhang L-H, Yun K, Kim SJ. Antibacterial Efficiency of Graphene Nanosheets against Pathogenic Bacteria via Lipid Peroxidation. *The Journal of Physical Chemistry C* 2012, **116**(32)**:** 17280-17287.

31. Gurunathan S, Han JW, Dayem AA, Eppakayala V, Kim J-HJIjon. Oxidative Stress-Mediated

Antibacterial Activity of Graphene Oxide and Reduced Graphene Oxide in Pseudomonas Aeruginosa. 2012, **7:** 5901.

32. Nguyen VH, Kim B-K, Jo Y-L, Shim J-J. Preparation and Antibacterial Activity of Silver Nanoparticles-Decorated Graphene Composites. *The Journal of Supercritical Fluids* 2012, **72:** 28-35.

33. Krishnamoorthy K, Umasuthan N, Mohan R, Lee J, Kim S-J. Antibacterial Activity of Graphene Oxide Nanosheets. *Science of Advanced Materials* 2012, **4**(11)**:** 1111-1117.

34. Bykkam S, Rao K, Chakra CS, Thunugunta TJIJoAB, Research. Synthesis and Characterization of Graphene Oxide and Its Antimicrobial Activity Against Klebseilla and Staphylococus. 2013, **4**(1)**:** 142-146.

35. Yadav SK, Jung YC, Kim JH, Ko Y-I, Ryu HJ, Yadav MK*, et al.* Mechanically Robust, Electrically Conductive Biocomposite Films Using Antimicrobial Chitosan-Functionalized Graphenes. *Particle & Particle Systems Characterization* 2013, **30**(8)**:** 721-727.

36. Li C, Wang X, Chen F, Zhang C, Zhi X, Wang K*, et al.* The Antifungal Activity of Graphene Oxide–Silver Nanocomposites. *Biomaterials* 2013, **34**(15)**:** 3882-3890.

37. Ocsoy I, Paret ML, Ocsoy MA, Kunwar S, Chen T, You M*, et al.* Nanotechnology in Plant Disease Management: DNA-Directed Silver Nanoparticles on Graphene Oxide as an Antibacterial Against Xanthomonas Perforans. *ACS Nano* 2013, **7**(10)**:** 8972-8980.

38. Gurunathan S, Han JW, Dayem AA, Eppakayala V, Park M-R, Kwon D-N*, et al.* Antibacterial Activity of Dithiothreitol Reduced Graphene Oxide. *Journal of Industrial and Engineering Chemistry* 2013, **19**(4)**:** 1280-1288.

39. Chen J, Peng H, Wang X, Shao F, Yuan Z, Han H. Graphene Oxide Exhibits Broad-Spectrum Antimicrobial Activity Against Bacterial Phytopathogens and Fungal Conidia by Intertwining and Membrane Perturbation. *Nanoscale* 2014, **6**(3)**:** 1879-1889.

40. Barua S, Thakur S, Aidew L, Buragohain AK, Chattopadhyay P, Karak N. One Step Preparation of a Biocompatible, Antimicrobial Reduced Graphene Oxide–Silver Nanohybrid As a Topical Antimicrobial Agent. *RSC Advances* 2014, **4**(19)**:** 9777-9783.

41. Al-Thani RF, Patan NK, Al-Maadeed MA. Graphene Oxide As Antimicrobial Against Two Gram-Positive and Two Gram-Negative Bacteria in Addition to One Fungus. 2014.

42. Hui L, Piao J-G, Auletta J, Hu K, Zhu Y, Meyer T*, et al.* Availability of the Basal Planes of Graphene Oxide Determines Whether It Is Antibacterial. *ACS Applied Materials & Interfaces* 2014, **6**(15)**:** 13183-13190.

43. Yang X, Li Z, Ju E, Ren J, Qu X. Reduced Graphene Oxide Functionalized with a Luminescent Rare-Earth Complex for the Tracking and Photothermal Killing of Drug-Resistant Bacteria. *Chemistry – A European Journal* 2014, **20**(2)**:** 394-398.

44. Li J, Wang G, Zhu H, Zhang M, Zheng X, Di Z*, et al.* Antibacterial Activity of Large-Area Monolayer Graphene Film Manipulated by Charge Transfer. *Scientific Reports* 2014, **4**(1)**:** 4359.

45. Liu S, Hu M, Zeng TH, Wu R, Jiang R, Wei J*, et al.* Lateral Dimension-Dependent Antibacterial Activity of Graphene Oxide Sheets. *Langmuir* 2012, **28**(33)**:** 12364-12372.

46. Ristic BZ, Milenkovic MM, Dakic IR, Todorovic-Markovic BM, Milosavljevic MS, Budimir MD*, et al.* Photodynamic Antibacterial Effect of Graphene Quantum Dots. *Biomaterials* 2014, **35**(15)**:** 4428-4435.

47. Kellici S, Acord J, Ball J, Reehal HS, Morgan D, Saha B. A Single Rapid Route for the Synthesis of Reduced Graphene Oxide with Antibacterial Activities. *RSC Advances* 2014, **4**(29)**:** 14858-14861.

48. Mangadlao JD, Santos CM, Felipe MJL, de Leon ACC, Rodrigues DF, Advincula RC. On the Antibacterial Mechanism of Graphene Oxide (GO) Langmuir–Blodgett Films. *Chemical Communications* 2015, **51**(14)**:** 2886-2889.

49. He J, Zhu X, Qi Z, Wang C, Mao X, Zhu C*, et al.* Killing Dental Pathogens Using Antibacterial Graphene Oxide. *ACS Applied Materials & Interfaces* 2015, **7**(9)**:** 5605-5611.

50. Dutta T, Sarkar R, Pakhira B, Ghosh S, Sarkar R, Barui A*, et al.* ROS Generation by Reduced Graphene Oxide (rGO) Induced by Visible Light Showing Antibacterial Activity: Comparison with Graphene Oxide (GO). *RSC Advances* 2015, **5**(98)**:** 80192-80195.

51. Gupta DK, Rajaura RS, Sharma KJIJEST. Synthesis and Characterization of Graphene Oxide

Nanoparticles and Their Antibacterial Activity. 2015, **1**(1)**:** 16-24.

52. Grace ESC, Annamalai A, Ponmari G, Vani C, Rose A, Gunasekaran VJJNN. Cytotoxicity and Antibacterial Characteristics of Graphene-Oxide Nanosheets Toward Human Pathogens. 2015, **15:** 1-6.

53. Ye S, Shao K, Li Z, Guo N, Zuo Y, Li Q*, et al.* Antiviral Activity of Graphene Oxide: How Sharp Edged Structure and Charge Matter. *ACS Applied Materials & Interfaces* 2015, **7**(38)**:** 21571-21579.

54. Barbolina I, Woods CR, Lozano N, Kostarelos K, Novoselov KS, Roberts IS. Purity of Graphene Oxide Determines Its Antibacterial Activity. *2D Materials* 2016, **3**(2)**:** 025025.

55. Nanda SS, Yi DK, Kim K. Study of Antibacterial Mechanism of Graphene Oxide Using Raman Spectroscopy. *Scientific Reports* 2016, **6**(1)**:** 28443.

56. Hui L, Huang J, Chen G, Zhu Y, Yang L. Antibacterial Property of Graphene Quantum Dots (Both Source Material and Bacterial Shape Matter). *ACS Applied Materials & Interfaces* 2016, **8**(1)**:** 20-25.

57. Li R, Mansukhani ND, Guiney LM, Ji Z, Zhao Y, Chang CH*, et al.* Identification and Optimization of Carbon Radicals on Hydrated Graphene Oxide for Ubiquitous Antibacterial Coatings. *ACS Nano* 2016, **10**(12)**:** 10966-10980.

58. Karahan HE, Wei L, Goh K, Liu Z, Birer Ö, Dehghani F*, et al.* Bacterial Physiology is a Key Modulator of the Antibacterial Activity of Graphene Oxide. *Nanoscale* 2016, **8**(39)**:** 17181-17189.

59. Wang X, Lu P, Li Y, Xiao H, Liu X. Antibacterial Activities and Mechanisms of Fluorinated Graphene and Guanidine-Modified Graphene. *RSC Advances* 2016, **6**(11)**:** 8763-8772.

60. Chen J, Zhou G, Chen L, Wang Y, Wang X, Zeng S. Interaction of Graphene and Its Oxide with Lipid Membrane: A Molecular Dynamics Simulation Study. *The Journal of Physical Chemistry C* 2016, **120**(11)**:** 6225-6231.

61. Combarros RG, Collado S, Díaz M. Toxicity of Graphene Oxide on Growth and Metabolism of Pseudomonas Putida. *Journal of Hazardous Materials* 2016, **310:** 246-252.

62. Kromka A, Jira J, Stenclova P, Kriha V, Kozak H, Beranova J*, et al.* Bacterial Response to

Nanodiamonds and Graphene Oxide Sheets. *physica status solidi (b)* 2016, **253**(12)**:** 2481-2485.

63. Hou W-C, Lee P-L, Chou Y-C, Wang Y-S. Antibacterial Property of Graphene Oxide: the Role of Phototransformation. *Environmental Science: Nano* 2017, **4**(3)**:** 647-657.

64. Lu X, Feng X, Werber JR, Chu C, Zucker I, Kim J-H*, et al.* Enhanced Antibacterial Activity Through the Controlled Alignment of Graphene Oxide Nanosheets. *Proceedings of the National Academy of Sciences* 2017, **114**(46)**:** E9793.

65. Gao Y, Wu J, Ren X, Tan X, Hayat T, Alsaedi A*, et al.* Impact of Graphene Oxide on the Antibacterial Activity of Antibiotics Against Bacteria. *Environmental Science: Nano* 2017, **4**(5)**:** 1016-1024.

66. Qiu J, Wang D, Geng H, Guo J, Qian S, Liu X. How Oxygen-Containing Groups on Graphene Influence the Antibacterial Behaviors. *Advanced Materials Interfaces* 2017, **4**(15)**:** 1700228.

67. Zou F, Zhou H, Jeong DY, Kwon J, Eom SU, Park TJ*, et al.* Wrinkled Surface-Mediated Antibacterial Activity of Graphene Oxide Nanosheets. *ACS Applied Materials & Interfaces* 2017, **9**(2)**:** 1343-1351.

68. Marković ZM, Matijašević DM, Pavlović VB, Jovanović SP, Holclajtner-Antunović ID, Špitalský Z*, et al.* Antibacterial Potential of Electrochemically Exfoliated Graphene Sheets. *Journal of Colloid and Interface Science* 2017, **500:** 30-43.

69. Navya Rani M, Ananda S, Rangappa D. Preparation of Reduced Graphene Oxide and Its Antibacterial Properties. *Materials Today: Proceedings* 2017, **4**(11, Part 3)**:** 12300-12305.

70. Panda S, Rout TK, Prusty AD, Ajayan PM, Nayak S. Electron Transfer Directed Antibacterial Properties of Graphene Oxide on Metals. *Advanced Materials* 2018, **30**(7)**:** 1702149.

71. Zolezzi C, Ihle CF, Angulo C, Palma P, Palza H. Effect of the Oxidation Degree of Graphene Oxides on Their Adsorption, Flocculation, and Antibacterial Behavior. *Industrial & Engineering Chemistry Research* 2018, **57**(46)**:** 15722-15730.

72. Singh M, Bajaj NK, Bhardwaj A, Singh P, Kumar P, Sharma J. Study of Photocatalytic and Antibacterial Activities of Graphene Oxide Nanosheets. *Advanced Composites and Hybrid Materials* 2018, **1**(4)**:** 759-765.

73. Geng H, Wang T, Cao H, Zhu H, Di Z, Liu X. Antibacterial Ability, Cytocompatibility and Hemocompatibility of Fluorinated Graphene. *Colloids and Surfaces B: Biointerfaces* 2019, **173:** 681-688.

74. Fallatah H, Elhaneid M, Ali-Boucetta H, Overton TW, El Kadri H, Gkatzionis K. Antibacterial Effect of Graphene Oxide (GO) Nano-Particles Against Pseudomonas Putida Biofilm of Variable Age. *Environmental Science and Pollution Research* 2019, **26**(24)**:** 25057-25070.

75. Yu C-H, Chen G-Y, Xia M-Y, Xie Y, Chi Y-Q, He Z-Y*, et al.* Understanding the Sheet Size-Antibacterial Activity Relationship of Graphene Oxide and the Nano-Bio Interaction-Based Physical Mechanisms. *Colloids and Surfaces B: Biointerfaces* 2020, **191:** 111009.

76. Zhao M, Shan T, Wu Q, Gu L. The Antibacterial Effect of Graphene Oxide on Streptococcus mutans. *Journal of nanoscience and nanotechnology* 2020, **20**(4)**:** 2095-2103.

77. Wei W, Li J, Liu Z, Deng Y, Chen D, Gu P*, et al.* Distinct Antibacterial Activity of a Vertically Aligned Graphene Coating Against Gram-positive and Gram-negative Bacteria. *Journal of Materials Chemistry B* 2020, **8**(28)**:** 6069-6079.

78. Qiu J, Liu L, Qian S, Qian W, Liu X. Why Does Nitrogen-Doped Graphene Oxide Lose the Antibacterial Activity? *Journal of Materials Science & Technology* 2021, **62:** 44-51.

79. Montes-Duarte GG, Tostado-Blázquez G, Castro KLS, Araujo JR, Achete CA, Sánchez-Salas JL*, et al.* Key Parameters to Enhance the Antibacterial Effect of Graphene Oxide in Solution. *RSC Advances* 2021, **11**(12)**:** 6509-6516.

80. Mann R, Mitsidis D, Xie Z, McNeilly O, Ng YH, Amal R*, et al.* Antibacterial Activity of Reduced Graphene Oxide. *Journal of Nanomaterials* 2021, **2021:** 9941577.

81. Nguyen HN, Rodrigues DF. Chronic Toxicity of Graphene and Graphene Oxide in Sequencing Batch Bioreactors: A comparative Investigation. *Journal of Hazardous Materials* 2018, **343:** 200-207.

82. Akhavan O, Ghaderi E. Escherichia Coli Bacteria Reduce Graphene Oxide to Bactericidal Graphene in a Self-Limiting Manner. *Carbon* 2012, **50**(5)**:** 1853-1860.

83. Yadav N, Dubey A, Shukla S, Saini CP, Gupta G, Priyadarshini R*, et al.* Graphene Oxide-Coated

Surface: Inhibition of Bacterial Biofilm Formation due to Specific Surface–Interface Interactions. *ACS Omega* 2017, **2**(7)**:** 3070-3082.

84. Dellieu L, Lawarée E, Reckinger N, Didembourg C, Letesson JJ, Sarrazin M*, et al.* Do CVD Grown Graphene Films Have Antibacterial Activity on Metallic Substrates? *Carbon* 2015, **84:** 310-316.

85. Kurantowicz N, Sawosz E, Jaworski S, Kutwin M, Strojny B, Wierzbicki M*, et al.* Interaction of Graphene Family Materials with Listeria Monocytogenes and Salmonella Enterica. *Nanoscale Research Letters* 2015, **10**(1)**:** 23.

86. Gurunathan S, Han JW, Dayem AA, Eppakayala V, Kim J-H. Oxidative Stress-Mediated Antibacterial Activity of Graphene Oxide and Reduced Graphene Oxide in Pseudomonas Aeruginosa. *Int J Nanomedicine* 2012, **7**(5901)**:** e14.

87. Chen, J, Wang X, Han H. A New Function of Graphene Oxide Emerges: Inactivating Phytopathogenic Bacterium *Xanthomonas oryzae pv. Oryzae*. *Journal of Nanoparticle Research* 2013, **15**(5): 1-14.

88. Barrios AC, Wang Y, Gilbertson LM, Perreault F. Structure–Property–Toxicity Relationships of Graphene Oxide: Role of Surface Chemistry on the Mechanisms of Interaction with Bacteria. *Environmental Science & Technology* 2019, **53**(24), 14679-14687.

89. Zhao F, Wang SC, Zhu ZL, Wang S, Liu F, Liu G. Effects of Oxidation Degree on Photo-Transformation and the Resulting Toxicity of Graphene Oxide in Aqueous Environment. *Environmental Pollution* 2019, **249**, 1106-1114.

90. Hou W C, Lee PL, Chou YC, Wang YS. Antibacterial Property of Graphene Oxide: the Role of Phototransformation. *Environmental Science: Nano* 2017, **4**(3), 647-657.
